# Structural and biochemical characterization of human Schlafen 5

**DOI:** 10.1093/nar/gkab1278

**Published:** 2022-01-17

**Authors:** Felix J Metzner, Elisabeth Huber, Karl-Peter Hopfner, Katja Lammens

**Affiliations:** Department of Biochemistry, Gene Center, Feodor-Lynen-Straße 25, 81377 München, Germany; Department of Biochemistry, Gene Center, Feodor-Lynen-Straße 25, 81377 München, Germany; Department of Biochemistry, Gene Center, Feodor-Lynen-Straße 25, 81377 München, Germany; Department of Biochemistry, Gene Center, Feodor-Lynen-Straße 25, 81377 München, Germany

## Abstract

The Schlafen family belongs to the interferon-stimulated genes and its members are involved in cell cycle regulation, T cell quiescence, inhibition of viral replication, DNA-repair and tRNA processing. Here, we present the cryo-EM structure of full-length human Schlafen 5 (SLFN5) and the high-resolution crystal structure of the highly conserved N-terminal core domain. We show that the core domain does not resemble an ATPase-like fold and neither binds nor hydrolyzes ATP. SLFN5 binds tRNA as well as single- and double-stranded DNA, suggesting a potential role in transcriptional regulation. Unlike rat Slfn13 or human SLFN11, human SLFN5 did not cleave tRNA. Based on the structure, we identified two residues in proximity to the zinc finger motif that decreased DNA binding when mutated. These results indicate that Schlafen proteins have divergent enzymatic functions and provide a structural platform for future biochemical and genetic studies.

## INTRODUCTION

The Schlafen (Slfn) family (1,2) belongs to the class of interferon-stimulated genes (ISGs) (3,4). Ten murine and six human Schlafen proteins have been identified, which can be categorized into three subgroups according to their size and domain architecture (1,2,4). The Schlafen proteins play roles in various cellular processes such as regulation of cell cycle (5,6), T cell quiescence (7–11), differentiation and proliferation (3,12–14), tumorigenesis (3,13,15), response to DNA damaging agents (16–23) and inhibition of viral replication (24–26).

All Schlafen family members share a highly conserved N-terminal core region of approximately 340 amino acids (1,2). Based on sequence similarity, this region has been predicted to contain a divergent AAA ATPase associated domain (27). In this work, the N-terminal region will be referred to as Schlafen core domain. While subgroup I Schlafen proteins consist of the Schlafen core domain only, subgroups II and III harbor a C-terminal linker domain of unknown function. In addition, subgroup III Schlafen proteins, such as human SLFN5 and SLFN11, possess an additional C-terminal domain with sequence homology to the family of SF1 DNA/RNA helicases (2). Subgroup I and II members are predominantly located in the cytoplasm, while subgroup III members were mainly detected in the nucleus (28).

The Schlafen protein family was initially described as a regulator in thymocyte maturation (1,2) and T cell quiescence in mice (7). The *elektra* mouse, which carries a point mutation in the *mSlfn2* gene, is characterized by immune deficiency and susceptibility to bacterial and viral infections. Several studies indicated that mSlfn2 might maintain the quiescent state of T-cells by promoting the expression of ‘quiescence’ genes and inactivation of genes required for proliferation or differentiation (7,10,29). Additionally, Fischietti *et al.* suggested that mSlfn2 plays a role in the transcriptional regulation of ISGs via the balancing of type I IFN-mediated activation of STAT1 and NF-κB (30).

The human Schlafen family member SLFN11 came into focus due to its ability to promote cancer cell death in response to DNA-damaging agents (16–17,20). The N-terminal domain of SLFN11 was shown to specifically cleave type II tRNAs (31). This leads to translational downregulation of a range of proteins such as Ataxia telangiectasia and Rad3-related (ATR), resulting in inhibition of the DNA damage-repair pathway (31). In addition, it sensitizes cancer cells to DNA targeting therapies by blocking replication in response to DNA damage sites (32). Chromatin opening and replication stalling seem to be independent of the downregulation of ATR (22), but depend on the C-terminal helicase domain (32). SLFN11 has been described as a promising biomarker in different types of cancer (16–17,33–39). Expression levels of SLFN11 can help to predict the response to a wide range of DNA-damaging anti-cancer agents across multiple cancer types. Hence, it was proposed that SLFN11 could have clinical applications for matching patients to DNA-damaging chemotherapies. More recently, SLFN11 was also reported to destabilize stalled replication forks (40) and was found to play a role as a regulator of protein quality control (41). Furthermore, SLFN11 specifically abrogates the replication of HIV by selectively inhibiting viral protein synthesis in HIV infected cells in a codon-usage dependent manner (24). Inhibition of viral protein translation is achieved by tRNA binding, which counteracts the virus-induced shift of the tRNA pool towards A/U (24). Furthermore, endoribonuclease activity has been observed for several Schlafen family members (42–44). C-terminally truncated rabbit and human SLFN14 (residues 1–400) were shown to bind ribosomes and cleave rRNA (43,44). Other studies showed cleavage of tRNA and rRNA by rat Slfn13 (rSlfn13), human SLFN13, mouse mSlfn8 and human SLFN12, suggesting a role in translational regulation (42,45). Recently, Garvie *et al.* presented that a tetrameric complex of two phosphodiesterases PDE3A and two SLFN12 molecules lead to a cytotoxic response in cancer cells (45). The small molecule DNMDP stabilized binding to PDE3A and increased the RNase activity of SLFN12, which was important for its cytotoxic function.

Although human SLFN5 is linked to tumorigenesis and is being investigated as a biomarker, its molecular functions are poorly characterized. Depending on the tumor type, SLFN5 can have inhibitory (4,46–47) or stimulatory (48,49) effects on tumorigenesis. A recent study showed an inhibitory effect on the transcription of the transcription factor ZEB1, which leads to the inhibition of the AKT signaling pathway in BRCA cells (47). The suppression is mediated by direct binding of SLFN5 to the promoter DNA of ZEB1 and requires the proteins C-terminal domain (50). Furthermore, SLFN5 suppresses cancer cell migration by inhibiting expression of the membrane-type metalloprotease MT1-MMP (51). Since SLFN5 interacts with STAT1 and functions not only as an ISG but also as a repressor of ISG transcription, the existence of a negative-feedback regulatory loop is speculated (48).

SLFN5 was shown to have antiviral activity upon infection with herpes simplex virus 1 (HSV-1). It represses HSV-1 transcription by binding to viral DNA and in turn, preventing RNA polymerase II from accessing viral promoters. However, in the presence of the viral E3 ubiquitin ligase ICP0, SLFN5 is ubiquitinated and subject to proteasomal degradation (26).

To understand the molecular mechanism of human SLFN5, we determined the cryo-EM structure of full-length human SLFN5 and the crystal structure of the SLFN5 core domain. The crystal structure, at a resolution of 1.8 Å, revealed a highly conserved zinc finger motif. We confirmed binding to various nucleic acid substrates and identified residues involved in nucleic acid binding in proximity to this motif. In contrast to sequence-based predictions, the SLFN5 core domain does not resemble an ATPase like fold and neither binds nor hydrolysis ATP. Despite the partial sequence conservation of the active site residues identified in rSlfn13, we did not observe ribonuclease activity for human SLFN5 towards type II tRNAs or DNA. Further, we compared the ribonuclease activity of mouse mSlfn2, mSlfn8 and human SLFN11 and structurally discussed the mSlfn2 *elektra* mutation. The cryo-EM map of full-length SLFN5 gives insights into the domain architecture and domain interfaces of group III Schlafen proteins. The full-length protein shows a high affinity to double-stranded DNA and binds ATP. Overall, we present a comprehensive structural and biochemical analysis of SLFN5 and group III Schlafen proteins and discuss similarities and differences throughout this diverse family.

## MATERIALS AND METHODS

### Protein expression and purification

The gene constructs encoding the N-terminal domain M1-D336 of human SLFN5, the N-terminal domain M1-D351 of murine mSlfn8 or full-length murine mSlfn2 were cloned into pET21a vector (Novagen) using NdeI/XhoI (Thermo) restriction enzymes. Site-directed mutagenesis for the generation of the SLFN5^1–336^ R271E and R326E and the mSlfn2 I135N mutations were performed using the Quickchange (Stratagene) protocol. *E. coli* Rosetta (DE3) cells containing the plasmids were grown at 37°C to an OD_600_ of 0.8. Protein expression was induced with 0.2 mM IPTG and cells were incubated over night at 18°C and harvested by centrifugation.

The cells expressing C-terminally His_6_-tagged SLFN5^1–336^ were resuspended in lysis buffer (50 mM HEPES, pH 8.0, 200 mM NaCl, 7 mM imidazole, 2 mM MgCl_2_, 4 mM β-mercaptoethanol) supplemented with a protease inhibitor cocktail (0.176 g/l phenylmethylsulfonyl fluoride, 0.316 g/l benzamidine hydrochloride, 1.372 mg/l pepstatin, 0.256 mg/l leupeptin, 0.2 mg/l chymostatin). The cells were disrupted by sonication and the insoluble cell debris was separated from the supernatant by centrifugation at 30 000 × g at 4°C for 30 min. The supernatant was applied onto a Ni-NTA column (Qiagen) and extensively washed with lysis and washing buffer (lysis buffer supplemented with 31 mM imidazole). The protein was eluted by applying elution buffer (50 mM HEPES, pH 8.0, 200 mM NaCl, 250 mM imidazole, 2 mM MgCl_2_, 4 mM β-mercaptoethanol). Further purification was performed by negative anion exchange chromatography (Q-HP, GE Healthcare), where SLFN5^1-336^ remained in the flow-through, and a subsequent Superdex 200 size-exclusion chromatography (GE Healthcare) by using the following buffer condition: 50 mM HEPES, pH 8.0, 200 mM NaCl, 2 mM MgCl_2_, 4 mM β-mercaptoethanol. The expression and purification of the SLFN5^1–336^ mutants were performed accordingly.

C-terminally His_6_-tagged mSlfn2 and mSlfn2 I135N were expressed and purified accordingly but the pH of the buffers was set to pH 7.5.

Cells expressing C-terminally His_6_-tagged murine Slfn8^1–351^ were harvested by centrifugation, resuspended in lysis buffer (50 mM Tris pH 8.2, 300 mM NaCl, 2 mM MgCl_2_, 10 mM imidazole) supplemented with protease inhibitor cocktail and disrupted by sonication. The lysate was cleared by centrifugation at 30 000 × g at 4°C for 30 minutes and the supernatant was incubated with pre-equilibrated Ni-NTA resin (Qiagen). The Ni-NTA resin was washed (50 mM Tris pH 8.2, 300 mM NaCl, 2 mM MgCl_2_, 15 mM imidazole) and the His_6_-tagged protein was eluted by applying 50 mM Tris pH 8.2, 300 mM NaCl, 2 mM MgCl_2_, 250 mM imidazole. The elution fractions were dialyzed against 20 mM Tris pH 8.2, 50 mM NaCl, 2 mM MgCl_2_, 0.5 mM DTT overnight. The dialyzed protein was further purified using anion exchange chromatography (HiTrap Q HP, GE Healthcare) and heparin chromatography (HiTrap Heparin HP, GE Healthcare). The protein was eluted by applying a linear gradient from 50 mM NaCl to 1 M NaCl in 20 mM Tris pH 8.2, 2 mM MgCl_2_, 0.5 mM DTT, respectively. For further purification, mSlfn8^1–351^ was applied onto a Superdex 200 16/60 column (GE Healthcare), pre-equilibrated with 20 mM Tris pH 8.2, 200 mM NaCl, 2 mM MgCl_2_, 0.5 mM DTT.

Prior to SEC and freezing, the proteins were concentrated with centrifugal concentrators (Amicon^®^ Ultra Centrifugal Filters, Merck). The purified proteins were flash frozen in liquid nitrogen and stored at -80°C until further use.

### Crystallization and structure determination of SLFN5^1–336^

1 μl of SLFN5^1–336^ purified from Rosetta cells and 1 μl of the reservoir solution were mixed and crystals were obtained by the hanging drop vapor diffusion method. Cubic crystals formed at a protein concentration of 7 mg/ml and 200 mM NaCl, 100 mM MES pH 5.8, 20% (v/v) PEG 2000 MME as reservoir conditions. Reservoir solution supplemented with 25% (v/v) ethylene glycol was used as cryo-protectant prior to flash freezing in liquid nitrogen. The concentration of SLFN5^1–336^ was set to 3.2 mg/ml for the needle shaped crystals and 0.1 M sodium acetate pH 5.0 and 1.5 M ammonium sulfate were used as reservoir. Reservoir solution supplemented with 25% (v/v) glycerol was used as cryo-protectant prior to flash freezing in liquid nitrogen.

Diffraction data were collected at the beamlines X06SA (PXI) and X06DA (PXIII) (Swiss Light Source, Paul-Scherrer Institute, Villigen, Switzerland) at 100 K. Data were integrated and scaled with XDS (52,53). Experimental phases were determined using a 3.2 Å Zn-SAD dataset from the needle shaped crystals measured at 1.28 Å wavelength. For the generation of the Zn^2+^-substructure and a poly-alanine model HySS (54), AutoSol (55) and Autobuild (56) within the Phenix (57) software and Chainsaw (58) within the CCP4 package (59) were used. The poly-alanine model was used as model for molecular replacement phasing of the 1.8 Å native dataset from the cubic crystals. This was done using Phaser (60,61) and the initial model was automatically rebuild with Autobuild (56). The final structure of SLFN5^1-336^ was solved at 1.8 Å by iterative refinement cycles in PHENIX (62) or Refmac (63,64). The structure was manually completed with COOT (65). Prior to model building and refinement, we randomly omitted 5% of the reflections for monitoring the free R value. Data collection and model statistics are stated in Supplementary Table S2. All figures were prepared using PyMOL Molecular Graphic Systems (version 2.0, Schrödinger, LLC) or UCSF ChimeraX (66).

### Right-angle light scattering measurement

The molecular weight of SLFN5^1–336^ was determined by size-exclusion chromatography (SEC)-coupled right-angle light scattering. The experiment was performed using a Superdex 200 10/300 Increase column (GE Healthcare), coupled to a right-angle laser static light scattering device and refractive index detector (Malvern/Viscotek). BSA was used to calibrate the system and the evaluation was performed using the OmniSEC software (Malvern/Viscotek).

### Nucleic acid substrates

DNA and RNA oligonucleotides were purchased from Metabion (Planegg, Germany) and Biomers (Ulm, Germany), respectively.

For the generation of double-stranded nucleic acid substrates, the single strands were mixed in an equimolar ratio, heated to 95°C for 10 min and slowly cooled down to room temperature. The nucleic acid substrates used in this work are summarized in Supplementary Table S3.

### Affinity measurement by fluorescence anisotropy

Initial protein dilutions (0, 1, 2, 3, 5, 10, 15, 20, 30, 40 and 60 μM if not stated otherwise) of SLFN5^1–336^ wild type and mutants were prepared in 2× assay buffer (100 mM HEPES pH 8.0, 100 mM NaCl) and then mixed with 6-FAM labeled DNA or RNA (at a final concentration of 100 nM) in a 1:1 (v/v) ratio. The reaction was incubated on ice for 30 min and the fluorescence anisotropy was subsequently measured at an excitation wavelength of 470 nm and an emission wavelength of 520 nm. The data sets were analyzed with Prism (GraphPad Software) and fit to a Hill model.

### [γ-^32^P] ATP hydrolysis assay

For the ATPase assay 5 μM SLFN5^1–336^ was incubated in presence or absence of 0.2 μM single-stranded 60-mer poly (dT) DNA in 50 mM Tris pH 7.5, 150 mM KCl, 5 mM MgCl_2_, 1.5 mM ATP and 10 nM [γ-^32^P] ATP (Hartmann Analytik, Germany) at 37°C for 0 or 60 min. For analysis, 1 μl of the reaction mixture was applied onto polyethyleneimine cellulose TLC plates (Sigma-Aldrich, Germany) and free phosphate was separated from ATP by thin layer chromatography in TLC running buffer (1 M formic acid, 0.5 M LiCl). [γ-^32^P] ATP was detected using a Typhoon FLA 9000 imaging system (GE healthcare).

### SLFN5^FL^ expression and purification

A construct encoding for full-length SLFN5 with an N-terminal double FLAG-tag and a HRV 3C cleavage site was purchased from GenScript. The construct was inserted into a pcDNA3.1 vector using a codon-optimized sequence for human expression systems. Expi293F cells (Thermo Fisher Scientific) were transfected with the SLFN5 expression vector using polyethylenimine (PEI, MW 40 000, Polysciences). Cells were cultured in Expi293 Expression Medium (Thermo Fisher Scientific) at 37°C and 5% CO_2_. After 72 h, the cells were harvested by centrifugation, resuspended in lysis buffer (50 mM Tris pH 7.1, 400 mM NaCl, 2 mM MgCl_2_) supplemented with protease inhibitor (0.18 g/l PMSF, 0.32 g/l benzamidine, 1.37 mg/l pepstatin A, 0.26 mg/l leupeptin, 0.2 mg/l chymostatin) and disrupted by sonication. The lysate was cleared by centrifugation at 30 000 × g at 4°C for 45 min and the supernatant was incubated with pre-equilibrated ANTI-FLAG M2 Affinity Gel (Sigma-Aldirch) for 60 min. The resin was washed with wash buffer (25 mM Tris pH 7.1, 250 mM NaCl, 2 mM MgCl_2_) and wash buffer supplemented with 1 mM ATP. After washing with buffer A (25 mM Tris pH 7.1, 120 mM NaCl, 2 mM MgCl_2_, 1 mM DTT), the protein was eluted in 4 × 1.1 ml elution buffer (buffer A supplemented with 0.2 mg/ml Flag-peptide) over 60 min. The eluate was loaded onto a HiTrap Heparin HP column (GE Healthcare) and the protein was eluted by a linear salt gradient (100 % buffer A to 100 % buffer B (25 mM Tris pH 7.1, 1 M NaCl, 2 mM MgCl_2_, 1 mM DTT) over 12 CV). The peak fractions were combined and applied onto a Superdex 200 5/150 column (GE Healthcare), pre-equilibrated with buffer A. The peak fractions were combined and flash frozen in liquid nitrogen. FLAG-tagged SLFN11 was purified following a similar protocol, with the difference, that the pH of the buffers was adjusted to pH 7.5.

### Cryo-EM grid preparation

Freshly purified SLFN5 was diluted to a final concentration of 3 μM using cryo-EM buffer (50 mM glycine pH 9, 50 mM NaCl, 2.5 mM MgCl_2_, 1 mM DTT). *n*-octyl-β-D-glucoside was added (0.045 %) and 4.5 μl of the sample was applied onto a glow discharged UltrAuFoil^®^ R2/2 holy gold grid. The sample was flash-frozen in liquid ethane, using an EM GP plunge freezer (Leica, 10°C and 90 % humidity).

### Cryo-EM data collection

The datasets were collected using a FEI Titan Krios G3 transmission electron microscope (300 kV), equipped with a GIF quantum energy filter (slit width 20 eV) and a Gatan K2 Summit direct electron detector. The data was automatically acquired using EPU (FEI). Three datasets were collected with 1765 (dataset I), 550 (dataset II) and 798 (dataset III) movies. Datasets II and III were collected at a tilt angle of 25°. All datasets were collected with a pixel size of 1.046 Å and 40 frames over 8 s. Dataset I and II were collected with a total electron dose of 41.2 e^–^/Å^2^ and dataset III with 40.9 e^–^/Å^2^. Defocus values ranging from –1.1 to –2.9 μm were applied.

### Cryo-EM data processing and 3D reconstruction

Motion correction of the movie frames was done using MotionCor2 (67). Unless stated otherwise, all subsequent processing steps were performed in cryoSPARC v3.2.0 (68) and the resolutions reported here are calculated based on the gold-standard Fourier shell correlation criterion (FSC = 0.143). The CTF parameters of the three datasets were determined using patch CTF estimation (multi) in cryoSPARC (v3.2.0). The exact processing scheme is depicted in Supplementary Figure S2. The data collection and refinement statistics are summarized in Supplementary Table S1. Initial particle picking was done using Blob picker on dataset III, yielding 607 362 particles, which were extracted with a box size of 256 px and a pixel size of 1.046 Å/px. The particles were subject to 2D classification and classes with clearly defined features were selected (43 089 particles). The selected particles were used as input for a Topaz train job, followed by particle extraction and 2D classification. The classes with clearly defined features yielded 102 102 particles, which were used as input for another round of Topaz train. The resulting Topaz model was used to pick on all three datasets. 592 223 particles were extracted from dataset I, 302 247 particles from dataset II and 344 426 particles from dataset III. Each particle set was subject to one round of 2D classification and ab-initio reconstruction. The resulting 192 301 particles from dataset I, 69 376 particles from dataset II and 140 963 particles from dataset III were combined (402 640 particles) and subject to another round of 2D classification and ab-initio reconstruction with five different classes. Three classes with 293 165 particles were selected and subject to heterogeneous refinement with four classes. The *ab-initio* reconstructions were used as input volumes for the heterogeneous refinement job. The class that showed the most defined features was selected (140 715 particles) and used for further refinement. The final resolution of the reconstruction after non-uniform refinement (69) was 3.44 Å.

### Model building

The SLFN5^1-336^ crystal structure as well as the AlphaFold v2.0 model (70) of the linker domain and the ATPase N-lobe were rigid body docked into the cryo-EM density using UCSF ChimeraX (66). Model building in COOT (65) and real space refinement in PHENIX (62) were performed iteratively using the 3.44 Å map.

### Purification of mononucleosomes

Canonical human histones were purchased from The Histone Source. For octamer assembly, the histones were resuspended in 7 M guanidinium chloride and mixed at a 1.2-fold excess of H2A and H2B. The mixture was dialyzed against 2 M NaCl for 16 h. The histone octamer was purified by size-exclusion chromatography using a Superdex 200 16/60 column (GE Healthcare). The Widom 601 DNA (71) with 80 bp of extranucleosomal DNA was amplified by PCR and purified by anion-exchange chromatography (Supplementary Table S3). DNA and histone octamer were mixed at a 1.1-fold excess of DNA in 2 M NaCl and diluted to 50 mM NaCl over 16 h at 4°C. Finally, the nucleosomes were purified by anion-exchange chromatography, dialyzed against 50 mM NaCl, concentrated and stored at 4°C.

### Electrophoretic mobility shift assay (EMSA)

EMSAs were conducted to analyze the interaction between SLFN5 and various nucleic acid substrates. Increasing amounts of SLFN5 were titrated to fluorescently labeled substrates (6-FAM or Cy5 labelled, 40 nM) in EMSA buffer (25 mM HEPES pH 7.0, 60 mM NaCl, 5 % glycerol, 2 mM MgCl_2_, 0.5 mM DTT) and incubated on ice for 30 minutes. The samples were analysed by native PAGE on 3–12 % acrylamide Bis-Tris gels (Invitrogen). The electrophoresis was performed in 1× NativePAGE Running Buffer (Invitrogen) at 4°C and 100 V for 120 min. The gels were visualized using a Typhoon FLA 9000 imaging system (GE healthcare). The images were analysed and integrated using GIMP v2.10.2 and ImageJ (72).

### Nuclease assay

The 6-FAM labeled nucleic acid substrate (tRNA_Ser_ or DNA) (50 nM) was incubated with the indicated Schlafen protein (250 nM) in nuclease buffer (25 mM Tris pH 7.3, 120 mM NaCl, 4 mM MgCl_2_, 1 mM DTT) at 37°C for 45 min. Where indicated, MnCl_2_ (2 mM) or EDTA (10 mM) was added. For the nuclease assay with DNA as a substrate, DNase I (Thermo Fisher Scientific) was used as positive control. The samples were analyzed on 15% denaturing polyacrylamide gels (Rotiphorese^®^ DNA sequencing system) in 1× TBE buffer. Gels were run at 270 V for 50 min and visualized using a Typhoon FLA 9000 imaging system (GE healthcare). The images were analysed and integrated using GIMP v2.10.2.

### Nano differential scanning fluorimetry (nanoDSF)

Interaction of SLFN5 with nucleotides was analyzed using nanoDSF (Tycho NT.6, NanoTemper Technologies). Full-length SLFN5 (500 nM) was incubated with or without nucleotides (1 mM) in buffer A (25 mM Tris pH 7.1, 120 mM NaCl, 2 mM MgCl_2_, 1 mM DTT) for 15 min on ice. The samples were loaded into glass capillaries and the internal fluorescence at 330 nm and 350 nm was measured while a thermal ramp was applied. The internal Tycho NT.6 software was used for data analysis, smoothing and calculation of derivatives. For SLFN5^1- 336^, SLFN5^1- 336^ R271E and SLFN5^1- 336^ R326E, a protein concentration of 2 μM was used and the pH was adjusted to pH 7.5.

### ATP hydrolysis assay

A fluorescence-based ATPase assay was conducted to determine the ATPase rate of SLFN5. SLFN5 (50 nM) was incubated with DNA or RNA substrates (150 nM) in assay buffer (25 mM Tris pH 7.5, 50 mM NaCl, 2 mM MgCl2, 0.1 mg/ml BSA, 1 mM DTT) at 25°C. In the assay, ATP (1 mM) hydrolysis is enzymatically coupled to the oxidation of NADH (0.1 mM) via phosphenolpyruvate (0.5 mM) by pyruvate kinase and lactate dehydrogenase (25 U/ml each, Sigma). Hexokinase from *Saccharomyces cerevisiae* was used as positive control (Sigma-Aldrich). The reaction volumes, of 50 μl each, were transferred to black non-binding 384-well plates (Greiner) and the fluorescence of NADH was measured using an Infinite M1000 PRO microplate photometer (TECAN). The reaction was monitored for 45 min (20 sec intervals) using an excitation wavelength of 340 nm and an emission wavelength of 460 nm.

## RESULTS AND DISCUSSION

### Cryo-EM structure of full-length SLFN5

**Figure 1.**
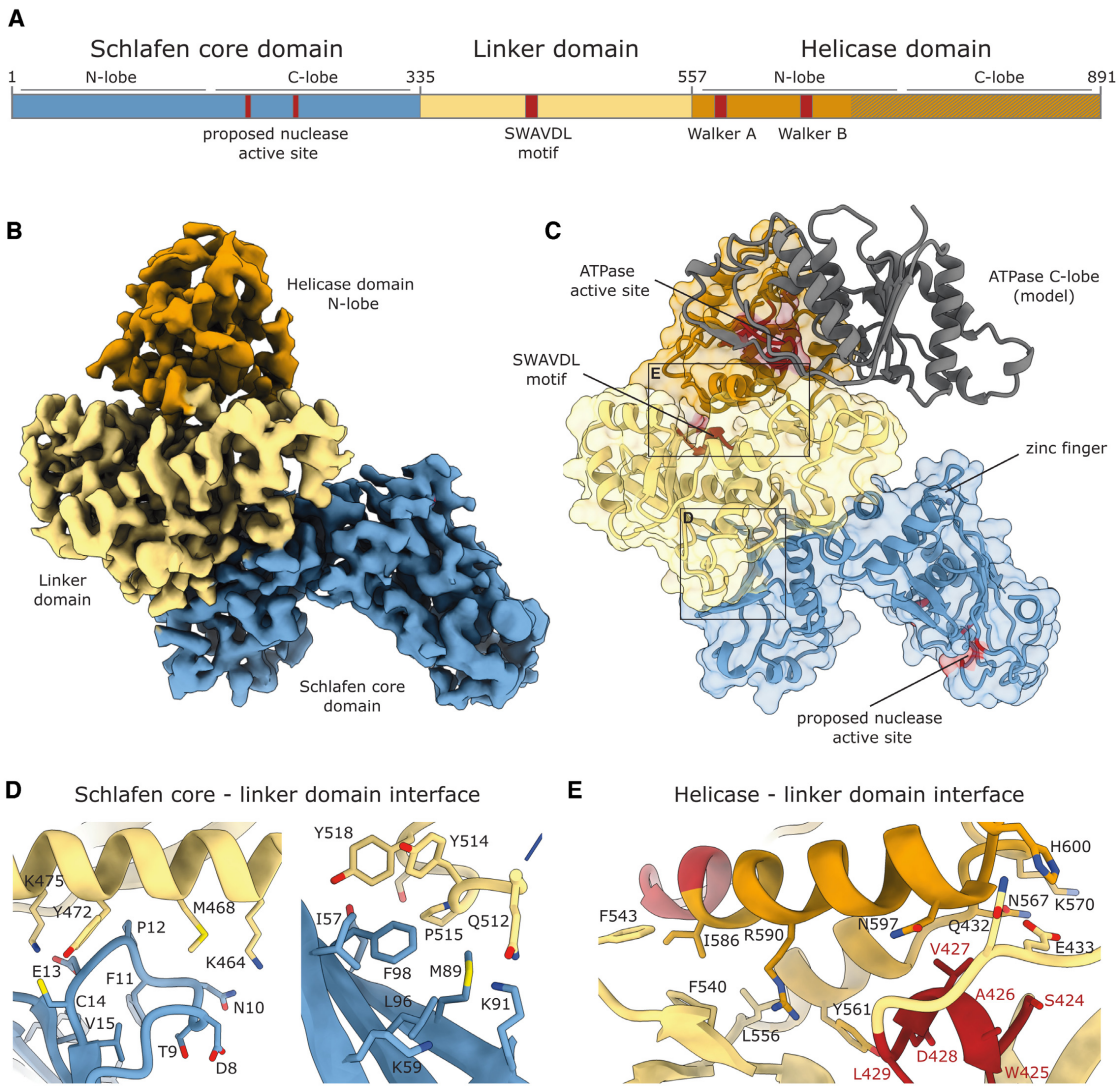
Structure of full-length SLFN5. (**A**) Domain architecture of Slfn5. (**B**) Cryo-EM reconstruction of full-length Slfn5. The Slfn core domain is depicted in blue, the linker domain in yellow and the helicase N-lobe in orange. (**C**) Ribbon and surface representation of Slfn5. Colored according to (A) with indicated motifs depicted in red. A model of the helicase C-lobe, as calculated by AlphaFold (70), is depicted in gray. (**D**) Detailed views of the interface between the linker and Schlafen core domain. (**E**) Detailed view of the interface between the linker and helicase domain N-lobe. The SWAVDL and Walker A motifs are colored in red.

**Figure 2.**
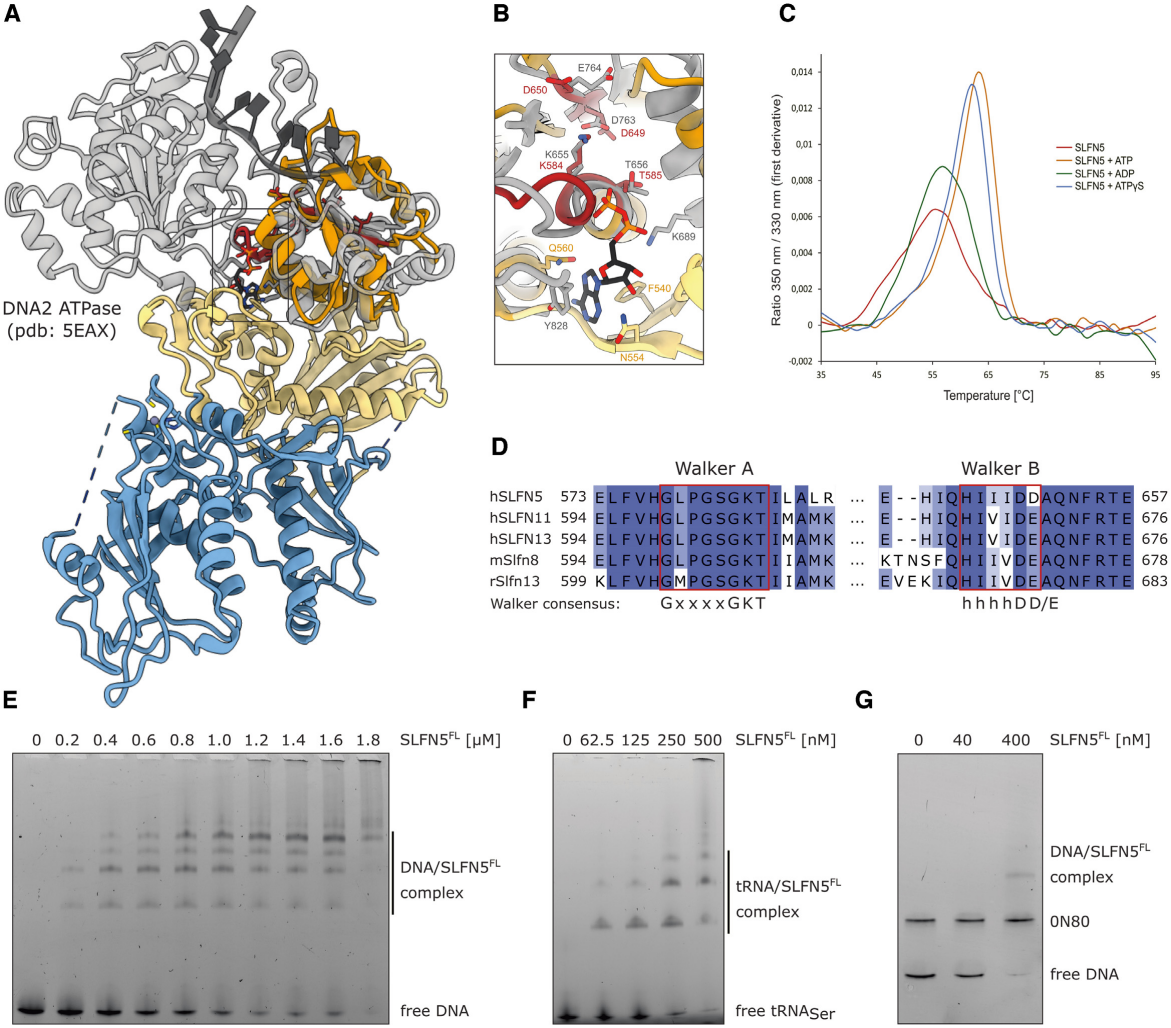
Biochemical characterization of full-length SLFN5. (**A**) Comparison of SLFN5 and the ATPase domain of DNA2 (gray) bound to ADP and ssDNA (only ATPase domain shown, PDB: 5EAX). (**B**) Superposition of ADP bound ATPase active site of DNA2 (gray) and the nucleotide free active site of SLFN5 (orange). The nucleotide and active site residues are displayed as sticks. (**C**) NanoDSF measurements of SLFN5 in presence of different nucleotides or without nucleotide. (**D**) Multiple sequence alignment of Walker A and B motifs of selected human, mouse and rat subgroup III Schlafen family members. Walker A and B motifs are highlighted in red. **(E)** Interaction of SLFN5 with 60 bp DNA monitored by electrophoretic mobility shift assay. (**F**) Interaction of SLFN5 with tRNA_Ser_ monitored by electrophoretic mobility shift assay. (**G**) Competition interaction analysis between SLFN5 and 0N80 nucleosome or 227 bp DNA monitored by electrophoretic mobility shift assay.

As a subgroup III Schlafen family member (2), SLFN5 possess a tripartite domain architecture (101 kDa). The N-terminal Slfn core domain (residues 1–336) is followed by a linker domain (residues 337–552) and a C-terminal helicase/ ATPase domain (residues 553–891). To allow for the biochemical and structural characterization of the full-length protein, a purification strategy was established. A human expression system was chosen, as bacterial expression did not yield soluble protein. Purification via the N-terminal FLAG-tag followed by heparin-affinity chromatography yielded protein of high purity (Supplementary Figure S1A, C). Analytical size exclusion chromatography showed a single peak with the elution volume approximately corresponding to a monomer (Supplementary Figure S1B). To gain insights into the structural organization of subgroup III Schlafen proteins, we employed single particle cryo electron microscopy (cryo-EM). The full-length SLFN5 protein was vitrified in the absence of nucleotides. Due to an orientation bias of the particles, the data was partially acquired at a tilt angle of 25°. A 3.5 Å cryo-EM density was calculated, giving insights into the overall domain arrangement (Figure 1A, B, Supplementary Figure S2A–F, Supplementary Table S1, Movie S1). A majority of the protein could be resolved (residues 3–684) with exception of the C-terminal ATPase lobe (residues 685–891), which is likely due to its flexibility (Figure 1C). Residues 143–165 are not resolved, indicating an unordered loop. The most N-terminal loop (residues 7–13) is shifted towards a hydrophobic patch of the N-lobe (Ile57, Leu96, Phe98), resulting in hydrophobic interaction via a semiconserved aromatic residue (Phe11) (Figure 1D). Furthermore, this loop contributes to interaction with the SLFN5 linker domain via a salt bridge between Glu13 and Lys475. The N-lobe of the Slfn core forms a second large interface with the linker domain, including several hydrophobic (Ile57, Met89, Phe98, Val479, Tyr514, Pro515, Tyr518) and ionic interactions (Asp87 to Arg487) (Figure 1D). In addition, the loop connecting the Slfn core to the linker domain (residues 335–366) holds the two domains together. The globular linker domain exhibits a mixed α/β topology and connects the Slfn core and the ATPase domain. The highly conserved SWAVDL motif (residues 424–429) seals the hydrophobic core of the linker domain and interacts with the N-terminal lobe of the helicase (Figure 1E). Density for the C-terminal ATPase lobe was not observed. The data was acquired in the absence of nucleotides, suggesting that the missing density of the second ATPase lobe could be due to relative flexibility between the lobes. A model of the C-lobe of the helicase, as calculated by AlphaFold (70), does not cause any steric clashes (Figure 1C). The N-terminal helicase lobe is anchored to the linker domain by an α-helix (residues 561–568) (Figure 1E). Further residues that are involved in the interaction are Gln432 and Arg590 of the linker domain. Apart from these charged residues, the helicase–linker domain interface is mostly hydrophobic (Figure 1E). Additionally, the loop preceding the ATPase domain, interacts with the ATPase N-lobe in close proximity to the putative ATP binding site via Phe540 and Phe543 (Figures 1E, 2B). The N-terminal ATPase lobe is highly conserved and harbors the characteristic residues of Walker A and B motifs that are essential for ATP binding and hydrolysis (Supplementary Figure S3). We superimposed the N-terminal ATPase lobe of SLFN5 with the nucleotide and ssDNA bound structure of the related SF1 helicase DNA2 (PDB: 5EAX) (Figure 2A, B). This suggests an accessible ATP binding site and sufficient space for the second ATPase lobe to bind. Assuming a similar DNA binding mode as in DNA2, the DNA binding site would be located on top of the ATPase domain (Figure 2A). The electrostatic surface potential of SLFN5 (Supplementary Figure S4) illustrates that the side of the molecule with the zinc finger and proposed DNA binding site in the helicase domain is positively charged, whereas the proposed nuclease active site would be located at the opposite side of SLFN5 with no clear surface charge potential. The distance between the positively charged patch next to the zinc finger motif and the predicted DNA binding site of the helicase domain is ∼40–45 Å (Supplementary Figure S4).

In summary, we present the first structure of a full-length subgroup III Schlafen family member, giving new insights into the structural organization of this class of proteins.

### Characterization of full-length SLFN5

As a subgroup III Schlafen family member, SLFN5 possess a C-terminal domain with homology to SF1 DNA/RNA helicases (2). SLFN5 harbours all characteristic motifs of SF1 DNA/RNA helicases, including the Walker A and Walker B motifs, which are involved in ATP binding and hydrolysis (Figure 2D). While the Walker A motif is highly conserved throughout subgroup III Schlafen family members, the last residue of the Walker B motif of SLFN5 is an aspartate compared to glutamate (Figure 2D). We tested nucleotide binding in a thermal unfolding assay using nano differential scanning fluorimetry (nanoDSF) (Figure 2C). Addition of ATP or ATPγS led to a shift of the inflection point towards higher temperature, indicating nucleotide binding. ADP binding resulted in a weaker shift of the inflection temperature. This could indicate structural differences between the diverse nucleotide states. Despite ATP binding by SLFN5, we could not detect ATP hydrolysis in a fluorescence-based ATPase assay (Supplementary Figure S5A-D). Neither single- or double-stranded DNA nor RNA or tRNA led to a stimulation of the ATPase. This could indicate that none of the tested substrates can stimulate the ATPase or that an essential additional factor, e.g. interaction partner, is still missing.

Compared to SLFN5^1-336^, full-length SLFN5 showed an increase in affinity to double-stranded DNA as well as tRNA (Figure 2E, F). In an electrophoretic mobility shift assay with a 60 bp substrate, four distinct band shifts were visible with the second and fourth shift showing higher intensity (Figure 2E). This could hint towards a cooperative binding model. At high SLFN5 concentrations, the protein-DNA complex did not enter the gel, indicating either aggregation or formation of large complexes. In competitive shift assays with tRNA_Ser_ and dsDNA, SLFN5 showed comparable affinities to tRNA_Ser_ and 50 bp DNA, but a preference for 196 bp DNA over tRNA_Ser_ (Supplementary Figure S6A, B).

SLFN11 was previously shown to be a regulator of chromatin structure, leading to increased accessibility of promoter sites. This activity is ATPase dependent (73). Based on its similarity to SLFN11, we investigated whether SLFN5 could interact with nucleosomes, as a majority of the DNA in the nucleus is organized in nucleosomes. In a competition assay with 227 bp DNA and a 0N80 nucleosome that was assembled on the same sequence (147 bp nucleosomal DNA and 80 bp of extranucleosomal DNA) as substrates, SLFN5 showed a clear preference for free DNA over nucleosomes (Figure 2G).

### Structural framework of SLFN5^1–336^

**Figure 3.**
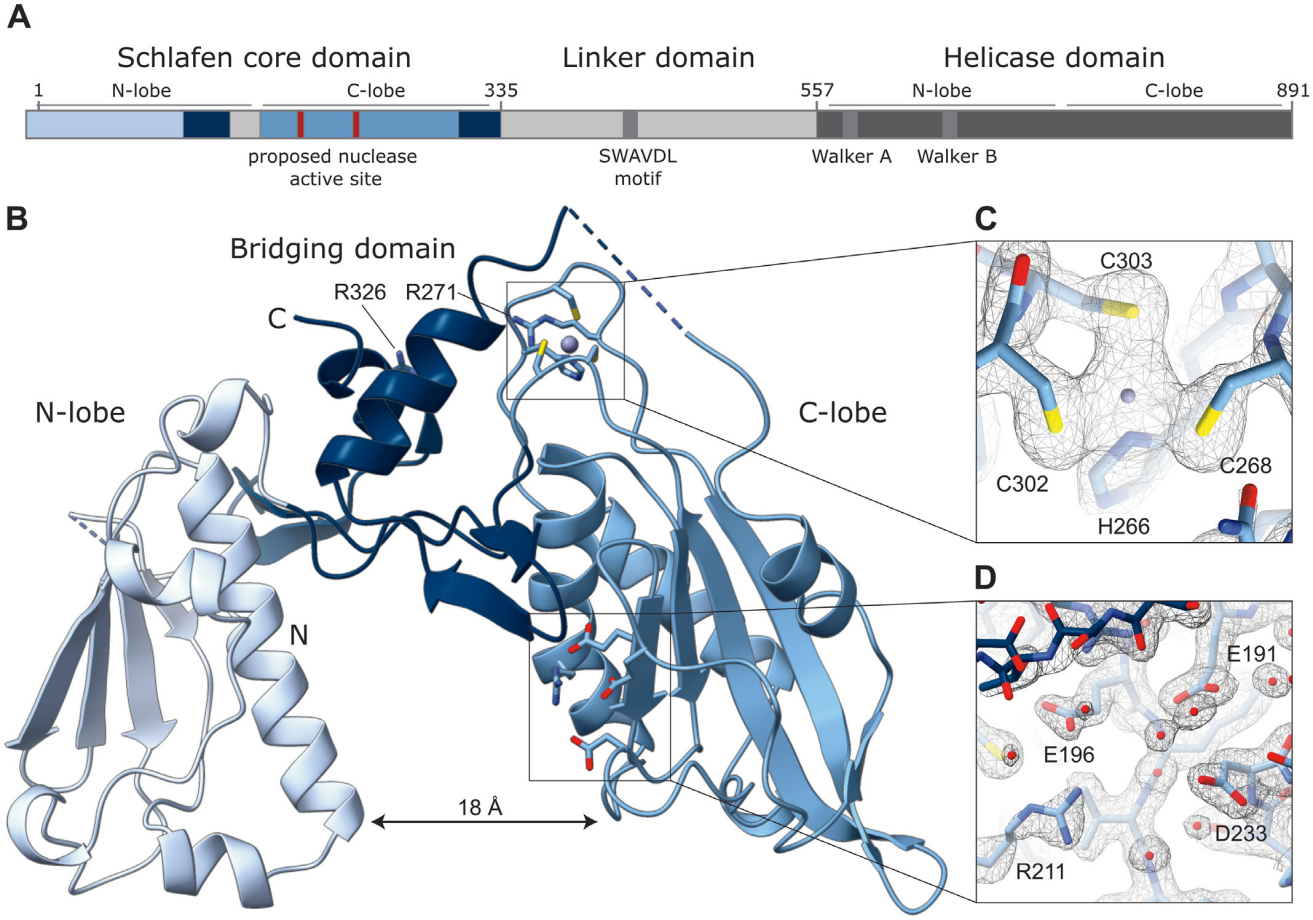
Structure of human SLFN5^1–336^ N-terminal domain. (**A**) Scheme of the domain architecture of human SLFN5. (**B**) Cartoon representation of SLFN5^1–336^ crystal structure solved in space group P2_1_. The residues of the zinc finger, the predicted nuclease active site and R271 and R326 are visualized as sticks. (**C**) Close-up view of the zinc coordinating residues (H266, C268, C302 and C303). The 2*F*_o_ – *F*_c_ electron density map is colored in black and contoured at σ = 1. (**D**) Electron density around the predicted active site residues (E191, E196, D233) indicate the 1.8 Å resolution. The 2*F*_o_ – *F*_c_ electron density map is colored in black and contoured at σ = 1.

To gain high resolution structural insights into human SLFN5, we crystallized the SLFN5 core domain (SLFN5^1–336^) (Figure 3A, B). The core domain (38.9 kDa) behaves as a monomer in solution, as determined by right angle light scattering (RALS) (Supplementary Figure S7A, B). SLFN5^1–336^ crystallized in the space groups P3_2_21 and P2_1_, diffracting to 3.2 and 1.8 Å, respectively (Supplementary Figure S8A, Figure 3B). The structure was determined by SAD using the intrinsically bound zinc ion and was refined to a resolution of 1.8 Å (Figure 3B, C and Supplementary Figure S8B). The detailed refinement statistics are summarized in Supplementary Table S2.

The SLFN5^1- 336^ structure depicts a horseshoe-like shape with a mixed α/β topology consisting of 10 α-helices and 14 β-sheets (Figure 3B, Supplementary Figure S8D). The approximate dimensions are 63 × 40 × 25 Å with the inner tunnel measuring 18 Å. The domain consists of an N-terminal and C-terminal lobe with respective bridging domain (Figure 3B). Each lobe consists of four α-helices and five β-sheets and each bridging domain of one helix and two sheets (Supplementary Figure S8D). The SLFN5^1–336^ structure comprises a zinc finger motif with the calculated anomalous map confirming the presence of a zinc ion (Figure 3C and Supplementary Figure S8B). The zinc ion is coordinated by a histidine and three cysteine residues (H266, C268, C302, C303) (Figure 3C) that are highly conserved throughout the entire Schlafen protein family (Supplementary Figure S3). In most parts, the crystal structure of SLFN5^1–336^ corresponds to the cryo-EM density.

The electron density for residues 145–168 could not be traced, indicating that this region consists of a flexible loop. We identified additional electron density close to the predicted active site, which could originate from a sulfate ion, as the protein was crystallized at an ammonium sulfate concentration of 1.5 M (Supplementary Figure S8C). The sulfate is coordinated by arginine 211 and via backbone interactions. As sulfates have been described to mimic phosphates of nucleic acids (74–76), this region could be a putative DNA binding or nuclease active site (Figure 3D).

The structure of SLFN5^1–336^ shows similarity to the published structure of the N-terminal domain of rat Slfn13 (rSlfn13^14–353^) (Supplementary Figure S8E) (42). However, the N-termini of the two structures adopt different conformations. The pseudo symmetry between the N- and C-lobes as reported for rSlfn13^14–353^ is broken in SLFN5^1–336^, as the sequential order of the secondary structure elements of each lobe differ slightly. SLFN5^1–336^ lacks a helix between α3 and α4 that is present in rSlfn13^14–353^. Moreover, the tunnel between the two lobes measures 18 Å in SLFN5^1–336^ but 23 Å in rSlfn13^14–353^. Taken together, the overall structure and the zinc finger motif of SLFN5^1–336^ are similar to rSlfn13^14–353^. However, differences between the secondary structural elements are evident.

### SLFN5^1-336^ nucleic acid binding properties

**Figure 4.**
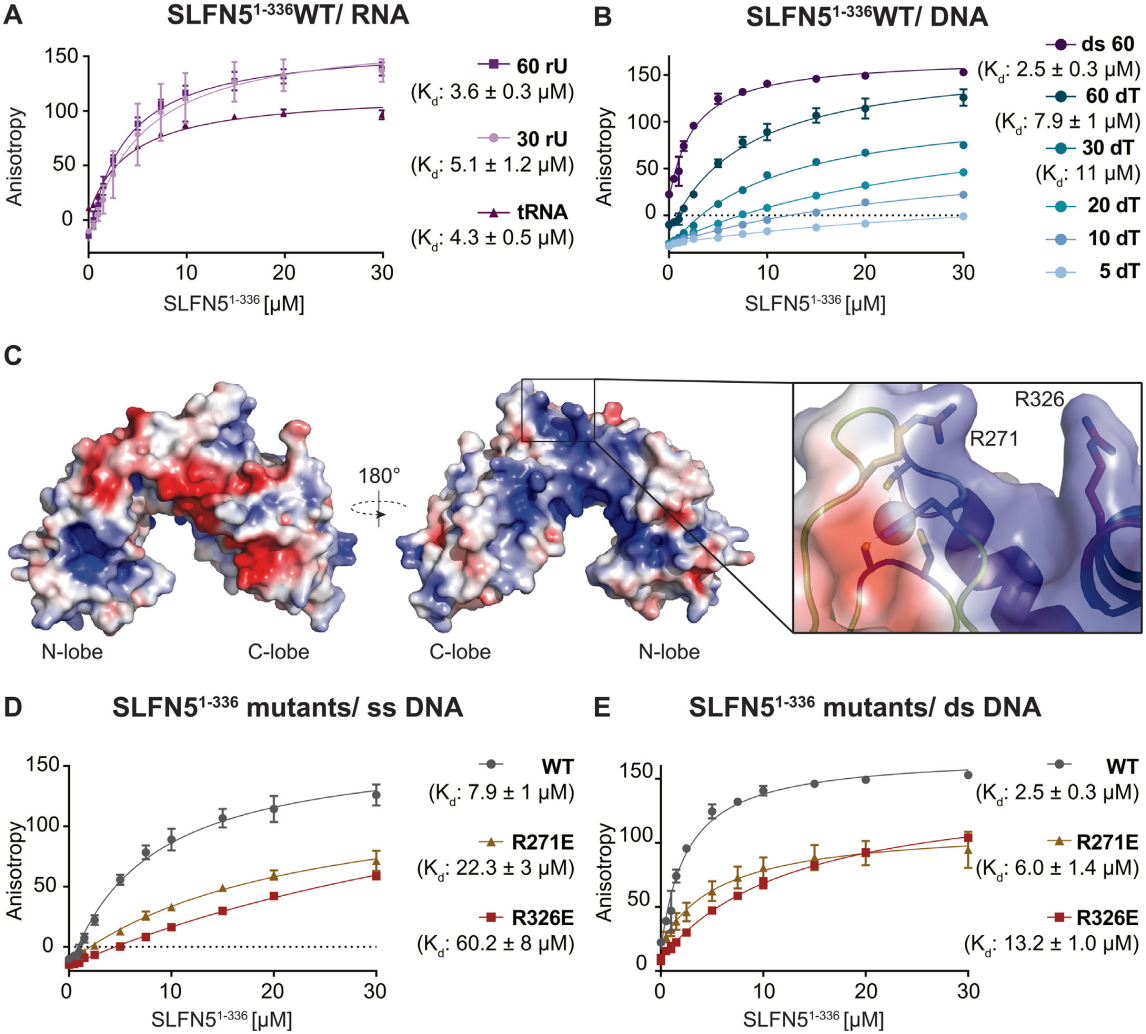
Analysis of SLFN5^1-336^ nucleic acid binding properties. (A, B) Fluorescence anisotropy assay to monitor the binding of SLFN5^1–336^ to different nucleic acid substrates. Different nucleic acid ligands are depicted in different colors. The data were fit to a 1 to 1 binding equation. Error bars represent the standard deviation from three independent experiments. (**A**) Binding of SLFN5^1–336^ to single-stranded 30-, 60-mer RNA and tRNA_Ser_. (**B**) Binding of SLFN5^1–336^ to single-stranded 5-, 10-, 20-, 30- and 60-mer poly d(T) DNA and double-stranded 60-mer DNA. (**C**) Electrostatic surface potential of SLFN5^1-336^ colored from red (–4kT/e) to blue (4kT/e) and close-up view of SLFN5^1–336^ R271 and R326, which are located in close proximity to the zinc finger. (D, E) Interaction of SLFN5^1–336^ and the charge reverse mutants SLFN5^1–336^ R271E and R326E to single-stranded 60-mer (**D**) and double-stranded 60-mer DNA (**E**) by the change in fluorescence anisotropy. The data were fit to a 1 to 1 binding equation. Error bars represent the standard deviation from three independent experiments.

Several members of the Schlafen family have been shown to interact with nucleic acids. While rSlfn13, human SLFN11 and rabbit Slfn14 are involved in tRNA or rRNA binding and processing (31,42,44), human SLFN5 has been shown to bind DNA, acting as a transcriptional regulator of ISGs (48). Therefore, we investigated the nucleic acid binding properties of SLFN5^1–336^ to various nucleic acid substrates (Supplementary Table S3) by fluorescence anisotropy experiments (FA) (Figure 4A, B).

The SLFN5 core domain revealed comparable affinities to all RNA types tested and binds tRNA_Ser_ with an equilibrium dissociation constant (K_d_-value) of 4.3 μM. Binding of SLFN5^1–336^ to 30- and 60-mer poly (rU) ssRNA resulted in *K*_d_ values of 5.1 and 3.6 μM, respectively (Figure 4A).

Furthermore, we analyzed SLFN5^1–336^ binding to various DNA substrates. The anisotropy measurements in the presence of single-stranded 5-,10-, 20- or 30-mer poly (dT) DNA (ssDNA) indicate low affinity to short ssDNA substrates (Figure 4B). With increasing DNA length, the affinity of SLFN5^1–336^ to single-stranded DNA was enhanced with *K*_d_ values of 11 μM and 7.8 μM for 30- and 60- mer poly (dT) ssDNA, respectively. The highest affinity was measured for 60-mer dsDNA with a *K*_d_ of 2.5 μM (Figure 4B).

In order to identify new residues involved in nucleic acid binding, besides the residues that were previously described (42), structure guided mutants were designed. Two arginine residues in close proximity to the zinc finger region emerge from the surface, suggesting an essential role of these residues in nucleic acid binding (Figure 4C). Based on this, two SLFN5^1–336^ mutants with substitutions of arginine to glutamate (R271E or R326E) were generated. Both mutants showed reduced binding to ssDNA, dsDNA and tRNA_Ser_ in anisotropy measurements (Figure 4D, E, Supplementary Figure S9A). The *K*_d_ values towards ssDNA, dsDNA and tRNA_Ser_ were increased compared to wild type SLFN5^1–336^, indicating that the identified residues R271 and R326 are involved in substrate binding. The inflection temperatures of the mutants are not decreased compared to wild type SLFN5^1-336^, indicating that the mutants are folded correctly (Supplementary Figure S9B). In summary, we could demonstrate a moderate binding preference of SLFN5^1–336^ for dsDNA and the importance of the zinc finger region for DNA binding.

### The Schlafen core domain has no ATPase activity

Based on sequence similarity, the N-terminal Schlafen domain was predicted to comprise a putative ATPase domain (27). Generally, the Walker A and B consensus sequences consist of GxxxxGK(T/S) (where x is any amino acid) and hhhhD(D/E) (where h is a hydrophobic amino acid) (77). In human SLFN14, the conserved aspartate residues D248 and D249 were predicted to be part of a Walker B motif (44). However, in this model, the Walker B motif would be inserted into a disrupted Walker A motif (Supplementary Figure S10A).

When comparing these residues in the crystal structures of SLFN5^1–336^ and rSlfn13 (Supplementary Figure S10B, C) to the active sites of other ATPases, no explicit structural similarity is evident. In SLFN5 and rSlfn13, the putative Walker A and B motifs are overlapping, whereas in functional ATPases, the motifs generally face each other to fulfill their function. Furthermore, the secondary structure elements in SLFN5^1–336^ and rSlfn13 are in a reverse order compared to known ATPases. The glycine-rich loop is preceded by a helix and followed by a β-sheet (Supplementary Figure S10B, C), while it is typically the other way around in known ATPases (77).

We tested nucleotide binding of SLFN5^1–336^ in a thermal unfolding assay using nano differential scanning fluorimetry (nanoDSF). None of the nucleotides tested (ADP, ATP or ATPγS) resulted in a change in the inflection temperature, suggesting that SLFN5^1–336^ does not bind to ATP (Supplementary Figure S10D). To test for ATP hydrolysis, SLFN5^1–336^ was incubated with radioactively labeled [γ-^32^P] ATP and the release of ^32^P_i_ was monitored by thin layer chromatography (TLC). However, regardless of the presence of 60-mer poly (dT) ssDNA, no significantly increased release of ^32^P_i_ was detected, indicating that no ATP hydrolysis took place (Supplementary Figure S10E).

The structural and biochemical data of SLFN5^1–336^ show that it does not resemble an ATPase-like fold, nor does it bind or hydrolyze ATP, confirming that the Schlafen core domain is not an ATPase.

### The Schlafen proteins share a similar fold, but differ in the predicted active site

Several Schlafen family members have been described to cleave and process RNAs. The N-terminal domain of rSlfn13 endonucleolytically cleaves tRNA_Ser_ in a Mg^2+^/Mn^2+^ dependent manner (42) and human SLFN11 was shown to cleave type II tRNAs (31). Therefore, we tested the nuclease activity of different Schlafen protein members on tRNA_Ser_.

**Figure 5.**
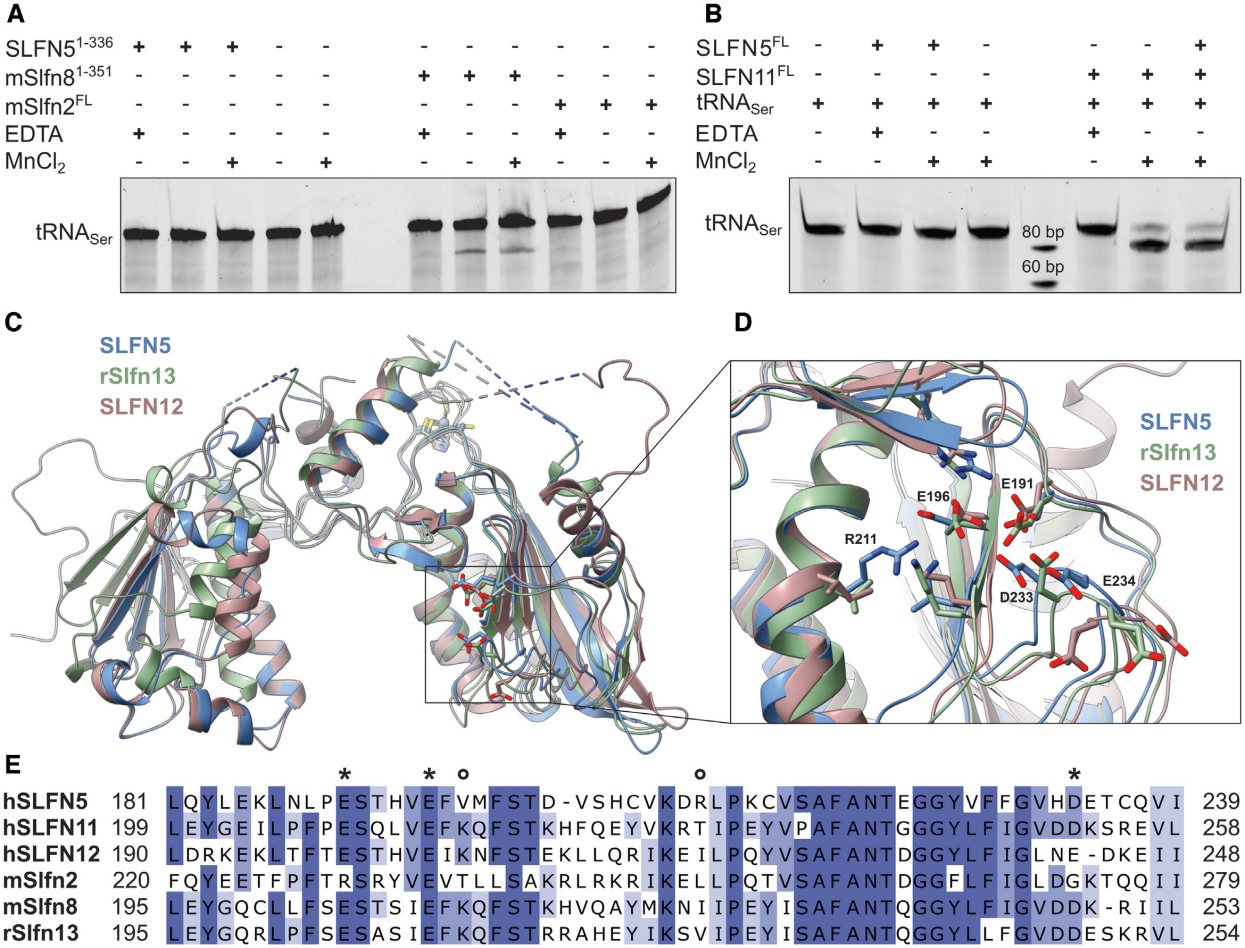
Comparison of the proposed nuclease active site between different Schlafen protein family members. (**A**) Endonuclease assay of SLFN5^1–336^, mSlfn8^1-351^ and mSlfn2^FL^ using tRNA_Ser_ as substrate. (**B**) Endonuclease assay of SLFN5 and SLFN11 using tRNA_Ser_ as substrate. (**C**) Overlay of the Slfn core domains of hSLFN5 (blue), rSlfn13 (green, PDB: 5YD0) and hSLFN12 (red, PDB: 7LRE). Residues of the proposed nuclease sites and the zinc finger are displayed as sticks. (**D**) Detailed view of the predicted nuclease site. Naming of the residues corresponds to SLFN5. (**E**) Multiple sequence alignment of the proposed active site regions of selected Schlafen proteins. The proposed catalytic residues are indicated (E/D = *, K/R = o)

SLFN5^1-336^ and full-length SLFN5 showed no nuclease activity on tRNA_Ser_, regardless of the presence of Mn^2+^ (Figure 5A, B). Murine Slfn8, a homologue of rat rSlfn13, was used as positive control. We confirm nuclease activity of mSlfn8^1-351^ on tRNA_Ser_ in a Mg^2+^/Mn^2+^ dependent manner, as the addition of EDTA prevented cleavage (Figure 5A). The cleavage pattern was similar to the one previously observed for rSlfn13 (42). Murine mSlfn2 showed no nuclease activity towards tRNA_Ser_ (Figure 5A), regardless if Mn^2+^ was present or not.

However, full-length SLFN11 exhibited Mn^2+^-dependent cleavage activity on tRNA_Ser_ (Figure 5B). The migration behavior of the cleavage product suggests the cleavage site to be located ∼10 nt from the 3’ end of the tRNA. This places the cut site between the acceptor arm and the T-arm of the tRNA. As SLFN5 binds, but does not cleave tRNA, we tested whether it could inhibit the tRNA cleavage activity of SLFN11. However, at equimolar concentrations, SLFN5 showed no inhibitory effect on tRNA cleavage by SLFN11 (Figure 5B). Furthermore, SLFN5^1-336^ showed no nuclease activity on ssDNA or dsDNA (Supplementary Figure S11).

Yang *et al.* identified a conserved three-carboxylate triad (E205, E210 and D248) that is responsible for the endonuclease activity of rSlfn13 (Figure 5C–E) (42). In human SLFN5^1–336^ the carboxylate motif, consisting of residues E191, E196 and D233, is conserved (Figure 5D). The additional density, which we interpreted as a sulfate ion in our crystal structure (Supplementary Figure S8C), is located in close proximity to the carboxylate motif, suggesting that this region could be involved in nucleic acid interaction, as sulfates are known to mimic the phosphate groups of nucleic acids (74–76). When comparing Schlafen 5 homologues of various species, the carboxylate triad exhibits little conservation. E196 (E210 in rSlfn13) is the only residue of the predicted carboxylate triad that shows a high degree of conservation, while E191 (E205 in rSlfn13) and D233 (D248 in rSlfn13) are only partially conserved (Supplementary Figure S3).

Since human SLFN12 and rSlfn13 have been shown to cleave rRNA and tRNAs, respectively (42,45), we superimposed the available structures to further investigate the active sites (Figure 5C, D). The superposition illustrates the similarities within their active site residues (Figure 5D). Additional to the carboxylate triad, both proteins harbor a lysine residue (K212 in rSlfn13 and K207 in SLFN12) in close proximity to the active site, which might be involved in the ribonuclease reaction. This lysine residue cannot be found in SLFN5 nor in mSlfn2. Instead, arginine R211 is involved in the coordination of the sulfate ion in the SLFN5^1–336^ structure (Supplementary Figure S8C). However, R211 in SLFN5 is also only partially conserved between different species.

The subgroup I Schlafen protein member mSlfn2 shows no cleavage activity on tRNA_Ser_ (Figure 5A). This is in line with the observation that murine mSlfn1, another Schlafen I protein subgroup member, lacks endonuclease activity as well (42). Out of the three proposed essential active site residues, only E235 (E196 in SLFN5) is present in mSlfn2 (Supplementary Figure S3). Instead, it harbors several positively charged amino acids (R247, R230, K274).

The lack of the lysine residue in SLFN5 and mSlfn2, which is conserved in cleavage proficient Schlafen family members mSlfn8, rSlfn13, hSLFN11 and hSLFN12, as well as the low conservation score for the residues of the three-carboxylate triad amongst Schlafen 5 proteins between different species, agree with the absence of nuclease activity in our assays.

In summary, our results indicate that even though the overall fold of the Slfn core domain is conserved, the enzymatic activity differs between the different Schlafen proteins.

### The *elektra* mutation could lead to misfolding and aggregation of the mSlfn2 protein

*Elektra* mice are highly immunodeficient due to a point mutation in the *mSlfn2* gene, which leads to a substitution of isoleucine 135 to asparagine (I135N) (7). In order to understand the influence of the *elektra* mutation on the protein structure, we generated an AlphaFold model of full-length mSlfn2 (70). I135 is located in the N-lobe of mSlfn2, surrounded by several hydrophobic residues forming a hydrophobic patch (Supplementary Figure S12A). These residues are highly conserved throughout all murine and human Schlafen members (Supplementary Figure 12B), indicating that the hydrophobic patch region is present in all Schlafen proteins. The introduction of a polar side chain into the hydrophobic patch by the I135N mutation might interfere with proper folding of the N-lobe, presumably resulting in aggregation. To support this hypothesis we expressed both wild type mSlfn2 and the I135N mutant in a bacterial expression system. We were able to solubly express and purify the wild type protein (Supplementary Figure S12C). On the other hand, the protein harboring the *elektra* mutation was insoluble, which might be a sign of aggregation.

## CONCLUSION

The Schlafen (Slfn) protein family belongs to the interferon-stimulated genes, which play key roles in immune defense and pathogen control (4). Although the Schlafen family members share many highly conserved sequence regions, their biological roles and enzymatic functions differ. While some family members such as rat Slfn13 and human SLFN11 influence the translation machinery (31,42), others, such as SLFN5, are involved in transcriptional regulation (26,47,50). In particular, the molecular mechanism of human SLFN5 in tumor control is not well understood.

Here, we present the cryo-EM structure of human full-length SLFN5 together with the high-resolution X-ray structure of the N-terminal core domain and biochemical data. The N-terminal domain is the common core domain of all Schlafen family members and supposedly involved in nucleic acid substrate recognition. The overall structural organization of the Schlafen core domain, including the zinc finger, is similar to rat Slfn13 (42). Nevertheless, they differ in some secondary structure elements and active site residues. Rat Slfn13 has been identified as an endoribonuclease with an active site consisting of a carboxylate triad in the C-lobe. However, the tRNA endoribonuclease activity could not be confirmed for human SLFN5, mSlfn1 or mSlfn2 by us and by others (42,78). In addition, the active site residues proposed by Yang *et al.* are not entirely conserved through all Schlafen family members (42). This confirms a divergent enzymatic function within the protein family. Despite performing nuclease activity assays with a variety of nucleic acids, we could not identify a substrate which is cleaved by SLFN5 or mSlfn2. However, we cannot exclude the necessity of additional factors for enzymatic activity. In order to understand the biochemical and enzymatic functions of the Schlafen core domain of SLFN5, we analyzed the nucleic acid binding properties. Our data show that the affinity of SLFN5^1–336^ to single-stranded DNA and RNA steadily increases in a length dependent manner from 20 bases up to 60 bases. The highest affinity was detected for a 60-mer double-stranded DNA, followed by 60-mer single-stranded RNA and tRNA_Ser_. Full-length SLFN5 binds double-stranded DNA with high affinity, which is in line with recent reports, showing SLFN5 to play a role in transcriptional regulation (26,50). The analysis of the electrostatic surface potential of the SLFN5 core domain revealed a positively charged patch. This region is in close proximity to the highly conserved zinc finger region and on the opposite site of the molecule compared to the previously proposed nuclease active site. To prove the involvement of this region in nucleic acid recognition, we mutated the residues R271 or R326 to glutamate, which decreased the binding affinity to DNA two to ten-fold and to tRNA_Ser_ two to five-fold, respectively. Thus, we identified an additional region involved in substrate binding. Furthermore, structural analysis as well as ATPase assays of SLFN5^1-336^ corroborated that the Schlafen core domain neither has the necessary ATPase motifs nor possess ATP hydrolysis activity, disproving earlier sequence-based predictions. We confirm a computational analysis, reporting that the Schlafen core domain is not an ATPase (79).

In contrast to the N-terminal core domain, our data demonstrate that full-length SLFN5 is able to bind ATP. Despite the fact that all necessary residues for an active ATPase exist in the C-terminal helicase domain of SLFN5, we could not observe ATP hydrolysis. The protein remained inactive in presence of different DNA and RNA substrates. We cannot exclude that additional cofactors, conformational rearrangements, interaction partners or substrates are needed to activate the enzymatic activity of the helicase domain.

The presented cryo-EM structure identified the linker domain of SLFN5 as a connector between the N-terminal SLFN5 core domain and the C-terminal helicase domain. The YPXSY motif (reidue 514–518) in the linker region as well as residue F98 involved in the interaction of the Schlafen core with the linker domain are highly conserved between subgroup II and III Schlafen family members including SLFN11 (Supplementary Figure S3). Moreover, the interface between the helicase and linker domain is conserved between the long Schlafen proteins, with the exception of Slfn14. This high degree of sequence conservation suggests that the identified domain organization and interfaces are preserved in both subgroups. However, Slfn14 seems to differ in the helicase - linker domain interface, which could indicate a divergent structural organization. This might be necessary for its function in ribosome degradation (43). In SLFN11, the clinically most relevant Schlafen protein, the domain interface motifs are conserved, suggesting a homologue structural arrangement. Recently, SLFN12 also came into focus of cancer research. It has been described that small molecule compounds, which stabilize complex formation between the phosphodiesterase PDE3A and SLFN12, cause selective cancer cell killing (45). The PDE3A–SLFN12 complex is formed by the interaction of the C-terminal extending PIR (phosphodiesterase interacting region) helix of SLFN12 with PDE3A. The structural comparison of SLFN5 with SLFN12 indicates that the PIR helix is only found in SLFN12 (Supplementary Figure S13A–D), excluding a similar interaction between SLFN5 and PDE3A. Based on sequence alignment and AlphaFold models (data not shown), the PIR helix cannot be found in subgroup III Schlafen proteins.

In summary, our study provides valuable insights into Schlafen subgroup III family members and will support the investigation of their diverse enzymatic functions.

## DATA AVAILABILITY

The coordinates and structure factors of the 1.8 Å and 3.2 Å SLFN5^1-336^ structures have been deposited in the Protein Data Bank under the accession codes 7Q3Z and 6RR9, respectively. The coordinates of the full-length SLFN5 structure have been deposited in the Protein Data Bank under the accession code 7PPJ. The SLFN5 reconstruction is available at the Electron Microscopy Data Bank under the EMDB accession code EMD-13581.

## Supplementary Material

gkab1278_Supplemental_Files
