# Supplementary material for: Structural and biochemical characterization of human Schlafen 5
Source: Nucleic Acids Res. 2022 Jan 17;50(2):1147–61. doi: 10.1093/nar/gkab1278 (PMC8789055; doi:10.1093/nar/gkab1278)
Supplement: gkab1278_Supplemental_Files [file gkab1278_supplemental_files.zip › Metzner_Huber_supplementary_information.pdf]

SUPPLEMENTARY DATA

Structural and biochemical characterization of human Schlafen 5

Felix J. Metzner, Elisabeth Huber, Karl-Peter Hopfner and Katja Lammens

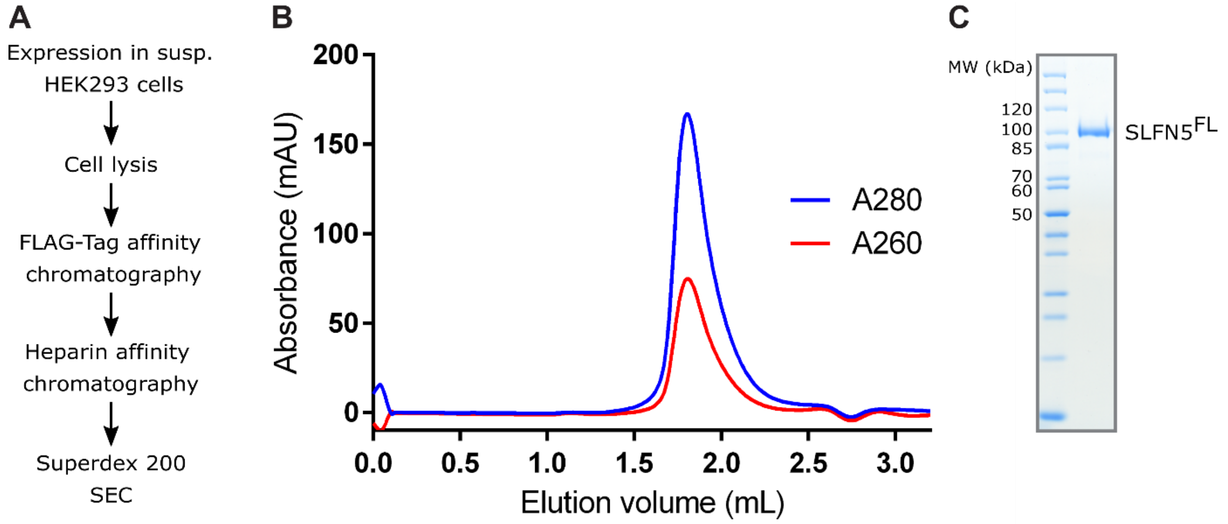

Supplementary Figure S1. Purification of full-length SLFN5. **(A)** Schematic of expression and purification of full-length SLFN5. **(B)** SEC elusion profile from a Superdex 200 5/150 column. Absorbance at 260 nm (red) and 280 nm (blue) is shown. **(C)** SDS-PAGE of purified full-length SLFN5 (stained with InstantBlue).

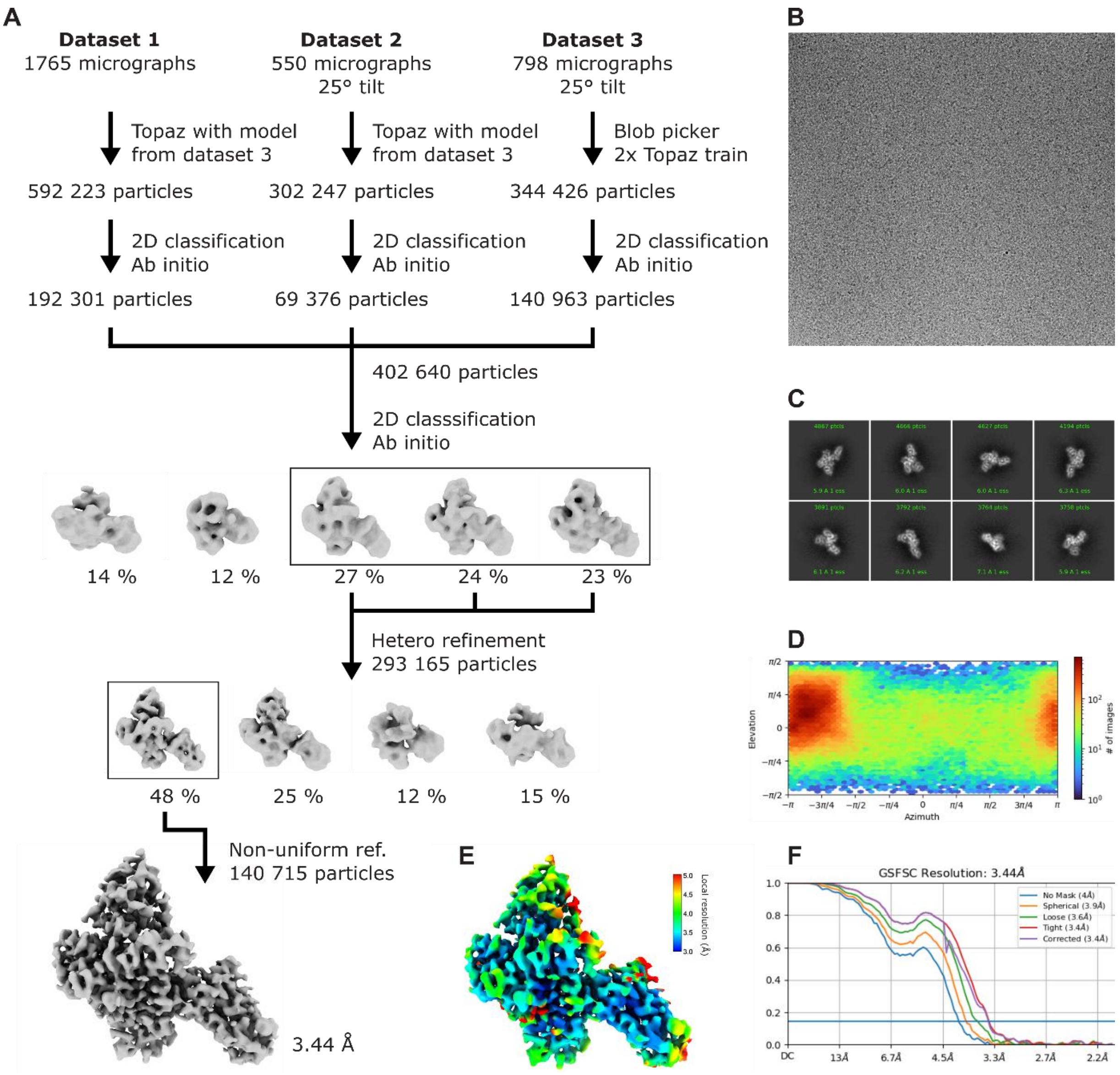

Supplementary Figure S2. Cryo-EM data analysis. **(A)** Cryo-EM data processing workflow of full-length SLFN5 using cryoSPARC v3.2.0 (1). **(B)** Representative micrograph of full-length SLFN5. **(C)** Representative classes of a 2D classification of the particles used for the final SLFN5 reconstruction. **(D)** Angular distribution of the particles used for the final SLFN5 reconstruction. **(E)** Visualization of local resolution calculated in cryoSPARC. Blue indicates higher resolution and red indicates lower resolution. **(F)** Gold-standard Fourier shell correlation (FSC) curves of the final SLFN5 reconstruction. The blue line indicates the 0.143 cutoff criterion, indicating a nominal resolution of 3.44 Å.

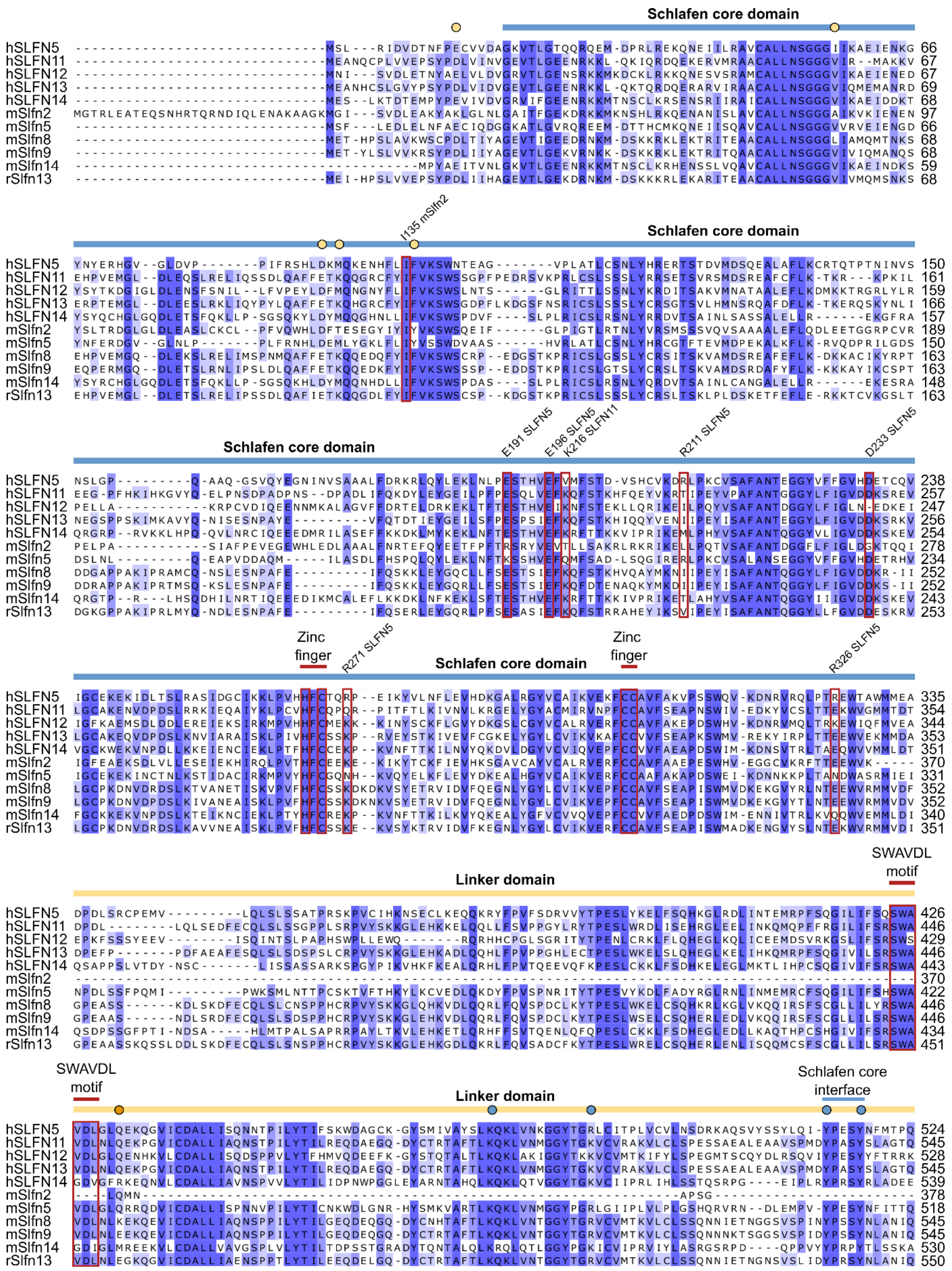

Schlafen core domain
I135 mSlfn2
Schlafen core domain
E191 SLFN5
E196 SLFN5
K216 SLFN11
R211 SLFN5
D233 SLFN5
Zinc finger
R271 SLFN5
Schlafen core domain
Zinc finger
R326 SLFN5
SWAVDL motif
Linker domain
SWAVDL motif
Linker domain
Schlafen core interface
hSLFN5
hSLFN11
hSLFN12
hSLFN13
hSLFN14
mSlfn2
mSlfn5
mSlfn8
mSlfn9
mSlfn14
rSlfn13

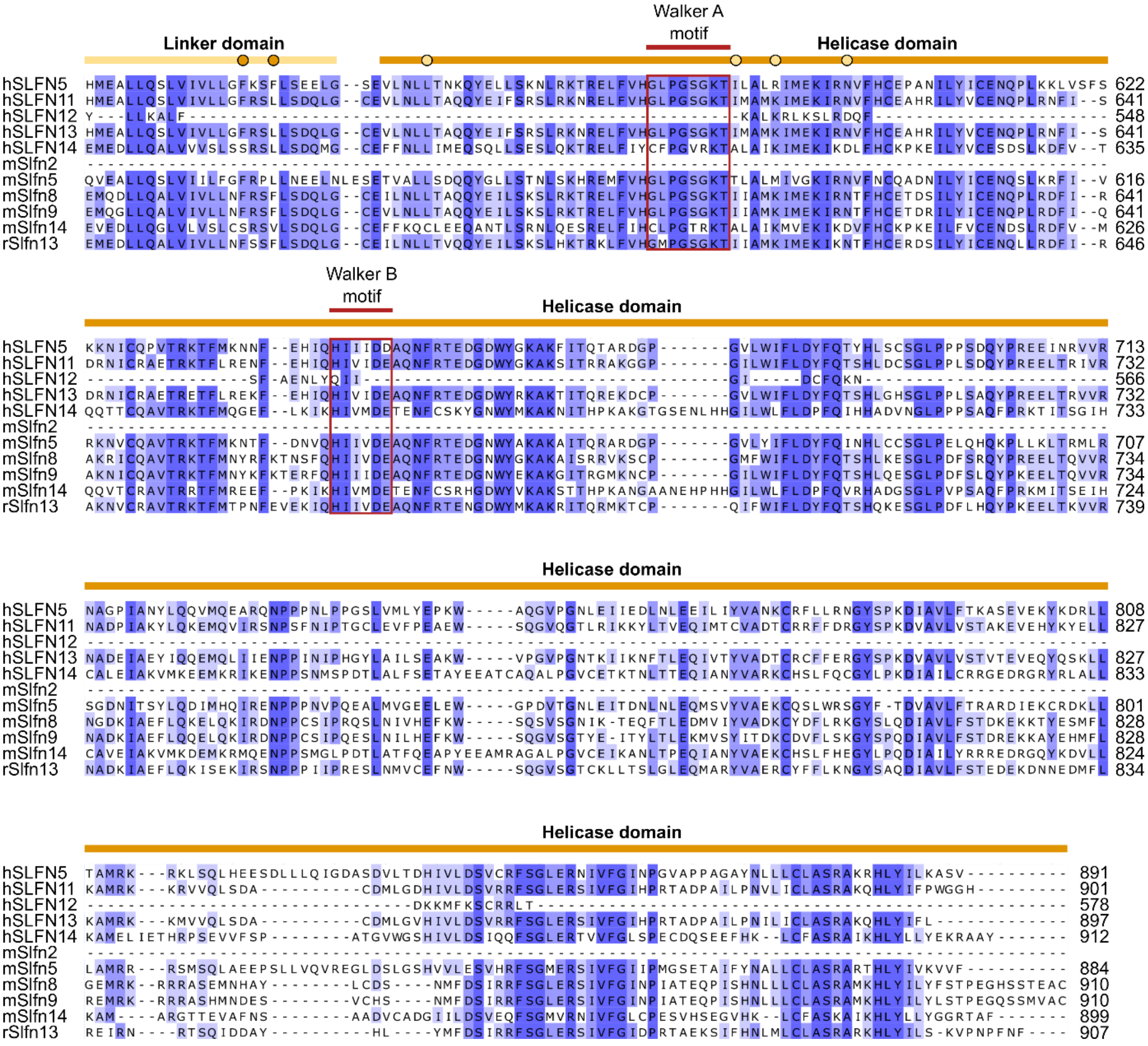

Supplementary Figure S3. Multiple sequence alignment of SLFN5 and selected Schlafen proteins from human (SLFN5, SLFN11, SLFN12, SLFN13, SLFN14), mouse (Slfn2, Slfn5, Slfn8, Slfn9, Slfn14) and rat (Slfn13). The alignment was calculated using MAFFT (2). Residues are colored according to percentage identity (dark blue = more conserved, white = less conserved). The Schlafen core domain is indicated in blue, the linker domain in yellow and the helicase domain in orange. The residues of the zinc finger, the SWAVDL motif, the Walker A and B motif and the position of the *elektra* mutation are highlighted in red. Residues involved in the Schlafen core domain – linker domain and the linker domain – helicase domain interaction are marked with circles.

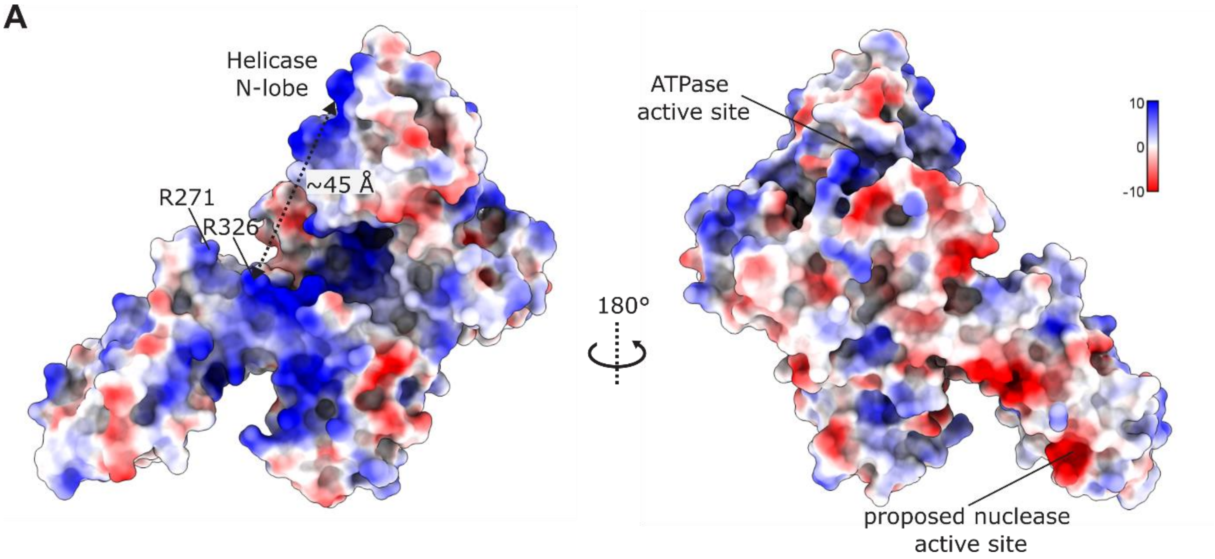

Supplementary Figure S4. Electrostatic surface potential of SLFN5. **(A)** Coulombic electrostatic surface potential of SLFN5 colored from red (-10 kcal/mol**e*) to blue (10 kcal/mol**e*). Values were calculated in UCSF ChimeraX (3). R271 and R326, the distance between R326 and the helicase domain and the putative nuclease and ATPase active sites are indicated.

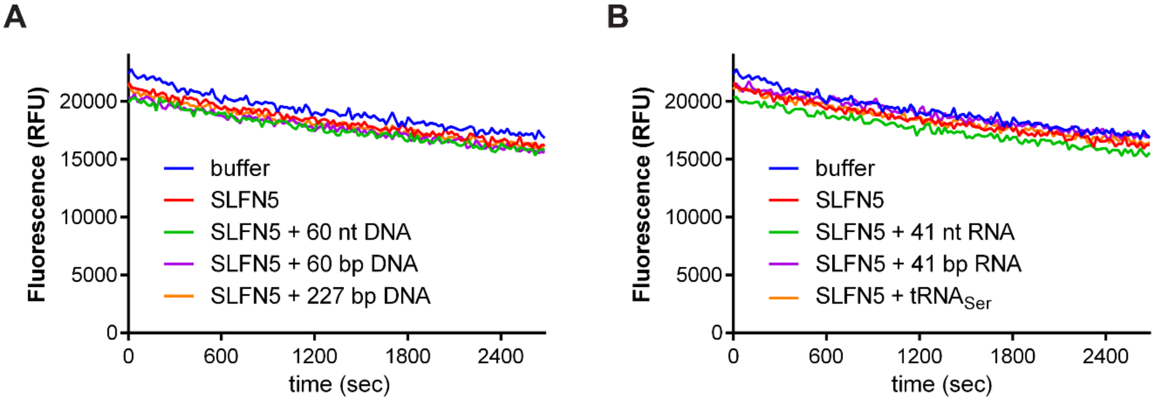

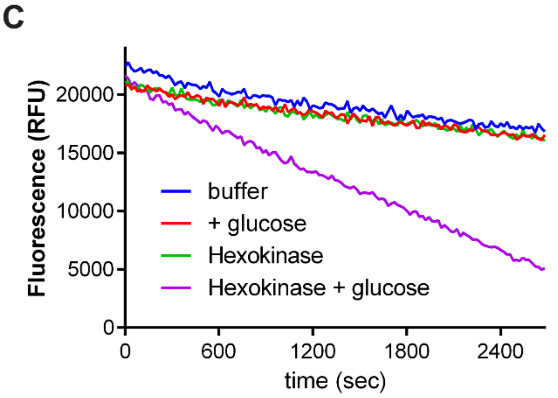

D

DNA fwd: TTTTTTTTTTTTTTTTTTTTTTTTTTTTTTTTTTTTTTTTTTTT
(60 nt) TTTTGCTTTTTTTTTT

DNA fwd: CTGGAGAATCCCGGTGCCGAGGCCGCTCAATTGGTCGTA
(227 nt) GCAAGCTCTAGCACCGCTTAAACGCACGTACGCGCTGTC
CCCCGCGTTTTAACCGCCAAGGGGATTACTCCCTAGTCTC
CAGGCACGTGTCAGATATATACATCCTGTGCATGTATTGAA
CAGCGACCTTGCCGGTGCCAGTCGGATAGTGTTCCGAGC
TCCCACTCTAGAGGATCCCCGGGTACCGA

RNA fwd: GACGGCCAUACCACCCUGAACGGCCCGAUCUUGUCUGA
(41 nt) UCC

$tRNA_{Ser}$: GUAGUCGUGGCCGAGUGGUUAAGGCGAUGGACUUGAA
AUCCAUUGUGGUUUCCCCGCGCAGGUUCGAAUCCUGCC
GACUACGCCA

Supplementary Figure S5. Fluorescence-based ATPase assay of SLFN5. Assay was performed in triplicates but a single replicate is shown due to visualization purposes. Data of buffer and SLFN5 is shown multiple times. **(A)** SLFN5 (50 nM) ATPase activity basal or in presence of ssDNA (60 nt, 150 nM) or dsDNA (60 bp and 227 bp, 150 nM). **(B)** SLFN5 (50 nM) ATPase activity basal or in presence of ssRNA (41 nt, 150 nM), dsRNA (41 bp, 150 nM) or $tRNA_{Ser}$ (150 nM). **(C)** Positive control: ATPase activity of hexokinase (1 nM) basal or in presence of glucose (200 µM). **(D)** Nucleotide sequences of substrates used in the ATPase assay (only forward sequences shown).

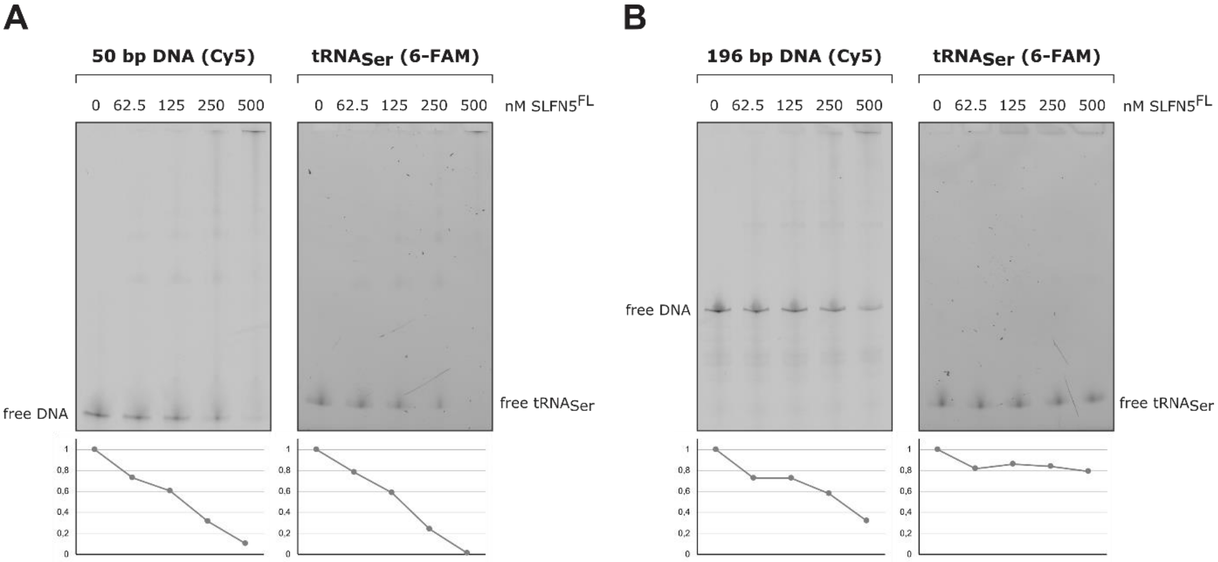

Supplementary Figure S6. Analysis of dsDNA and tRNA binding properties by electrophoretic mobility shift assay (EMSA) of SLFN5. **(A)** Competition EMSA of SLFN5 with Cy5 labeled 50 bp DNA and 6-FAM labeled $tRNA_{Ser}$ (20 nM each). The same gel was scanned twice, using the Cy5 (left) or FAM (right) channel. Bottom: Quantification of free DNA and tRNA bands using ImageJ (4). First lane was set to 1. **(B)** Competition EMSA of SLFN5 with Cy5 labeled 196 bp DNA and 6-FAM labeled $tRNA_{Ser}$ (20 nM each). The same gel was scanned twice, using the Cy5 (left) or FAM (right) channel. Bottom: Quantification of free DNA and tRNA bands using ImageJ. First lane was set to 1.

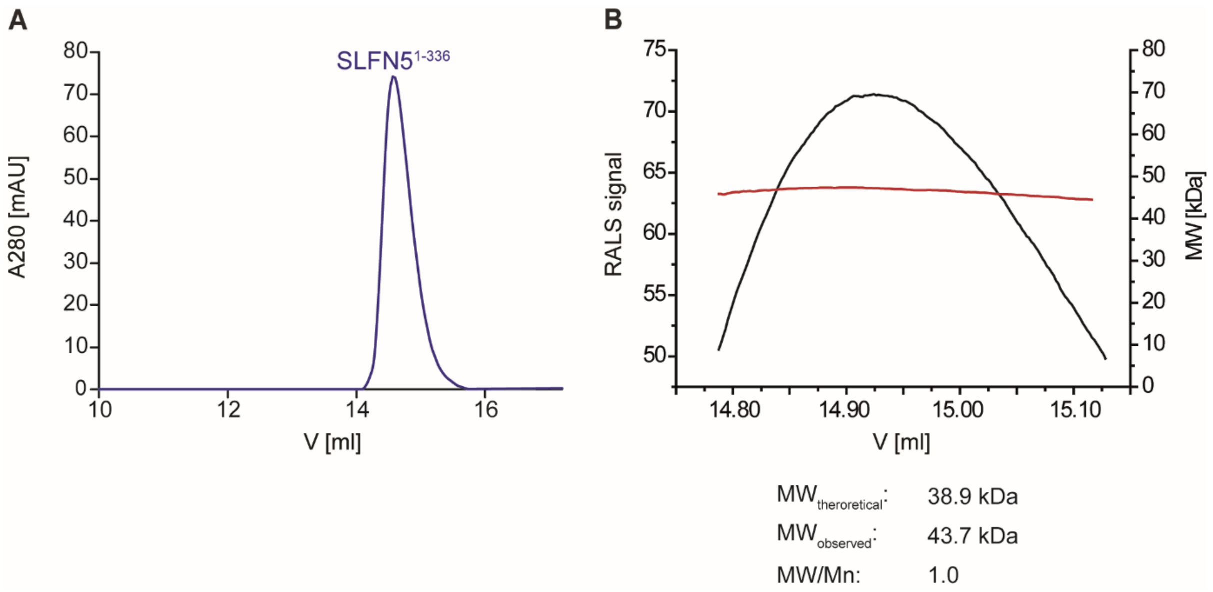

Supplementary Figure S7. Molecular weight determination of SLFN5$^{1-336}$ by SEC-RALS. **(A)** Analytical size exclusion chromatography using a Superdex 200 10/300 column. SLFN5$^{1-336}$ elutes in a single peak at approximately 15 ml. The blue line indicates the absorption at 280 nm. (**B)** Right-angle light scattering (RALS) coupled to SEC. The SLFN5$^{1-336}$ sample is monodisperse and the observed molecular weight was calculated to be 43.7 kDa, which corresponds to a monomer in solution. MW: molecular weight, Mn: number average molecular weight.

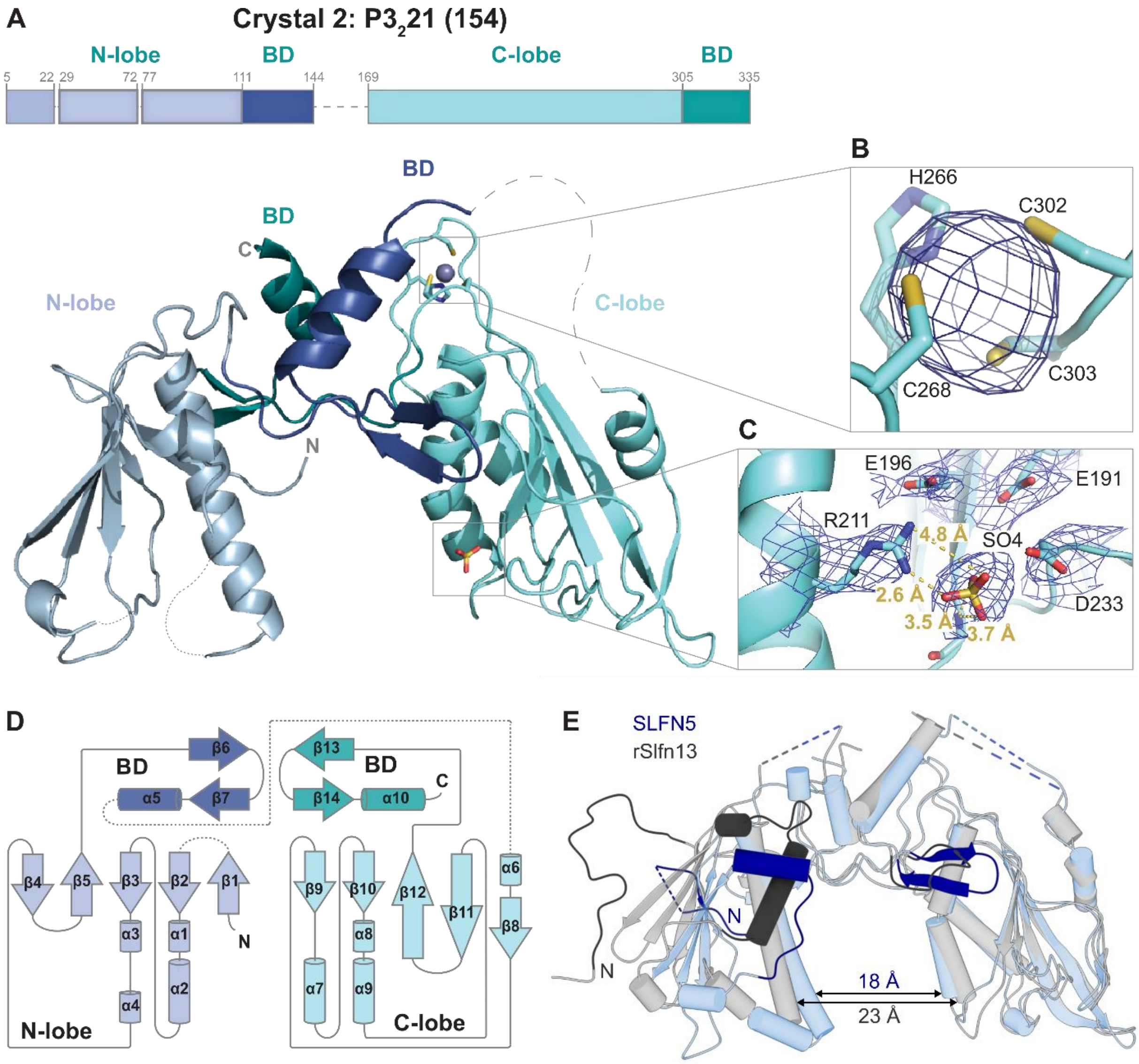

Supplementary Figure S8. Crystal structure of SLFN5$^{1\text{-}336}$ crystallized in space group P3$_2$21. **(A)** Schematic overview and structure of SLFN5$^{1\text{-}336}$ (P3$_2$21). N-lobe: light blue, N-bridging domain (BD): blue, C-lobe: cyan, C-BD: dark cyan. **(B)** Close-up view of the zinc finger motif. The anomalous electron density map is colored in dark blue and contoured at σ=5. **(C)** Close-up view of the bound sulfate and neighboring residues. The 2F$_0$-F$_c$ electron density map is colored in dark blue and contoured at σ=1. The interaction distances are indicated in yellow. **(D)** Secondary structure topology diagram of SLFN5$^{1\text{-}336}$. **(E)** Overlay of SLFN5$^{1\text{-}336}$ (blue) and rSlfn13$^{14\text{-}353}$ (gray, PDB: 5YD0). Selected regions showing structural differences are highlighted.

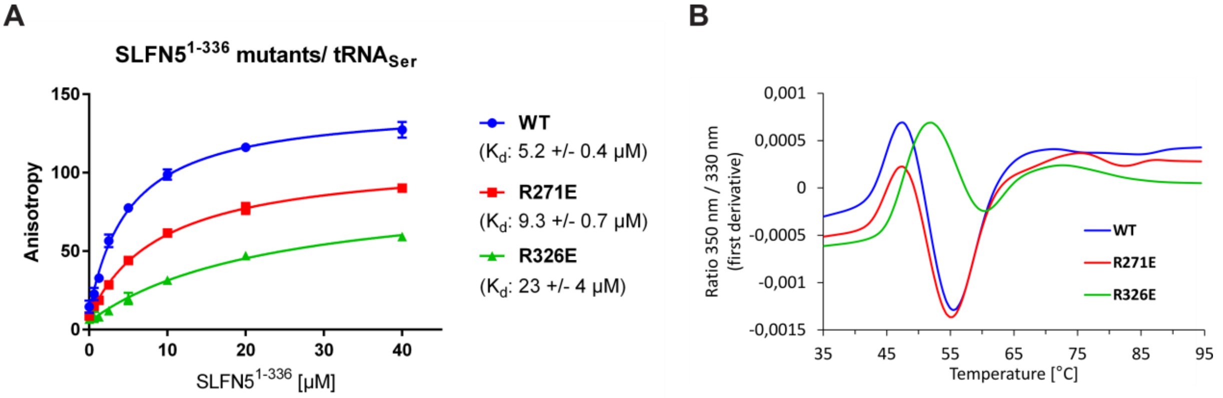

Supplementary Figure S9. Analysis of SLFN5$^{1\text{-}336}$ tRNA$_{Ser}$ binding properties. **(A)** Fluorescence anisotropy assay to monitor the binding of SLFN5$^{1\text{-}336}$ (blue), SLFN5$^{1\text{-}336}$ R271E (red) and SLFN5$^{1\text{-}336}$ R326E (green) to tRNA$_{Ser}$. The final protein concentrations were 0, 0.625, 1.25, 2.5, 5, 10, 20 and 40 µM. The data were fit to a 1 to 1 binding equation. Error bars represent the standard deviation from three experiments. **(B)** NanoDSF measurements of SLFN5$^{1\text{-}336}$ (blue), SLFN5$^{1\text{-}336}$ R271E (red) and SLFN5$^{1\text{-}336}$ R326E (green).

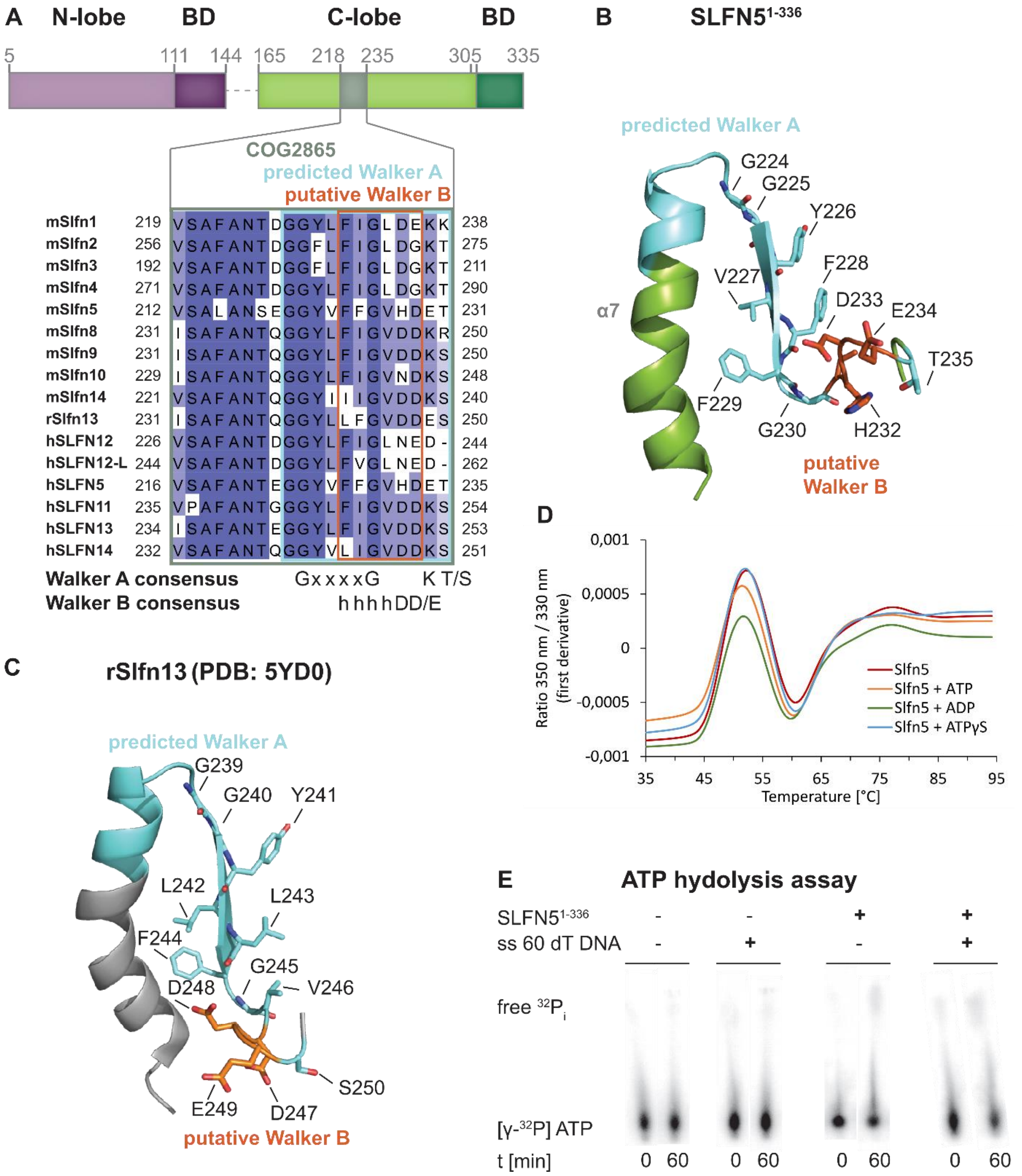

Supplementary Figure S10. Structural and functional analysis of the predicted ATPase site within the Slfn core domain. **(A)** Schematic overview of the domain organization of SLFN5$^{1\text{-}336}$ and multiple sequence alignment of the predicted Walker A/ Walker B motifs of murine and human Schlafen members. **(B-C)** Structural framework of the predicted Walker A/ Walker B motifs of SLFN5 (B) and rSlfn13 (PDB: 5YD0) (C). The predicted Walker A and B motifs are colored in aquamarine and orange, respectively. **(D)** NanoDSF measurements of SLFN5$^{1\text{-}336}$ in presence of different nucleotides or without nucleotide. **(E)** ATP hydrolysis assay of SLFN5$^{1\text{-}336}$ in presence or absence of a single-stranded 60-mer DNA substrate.

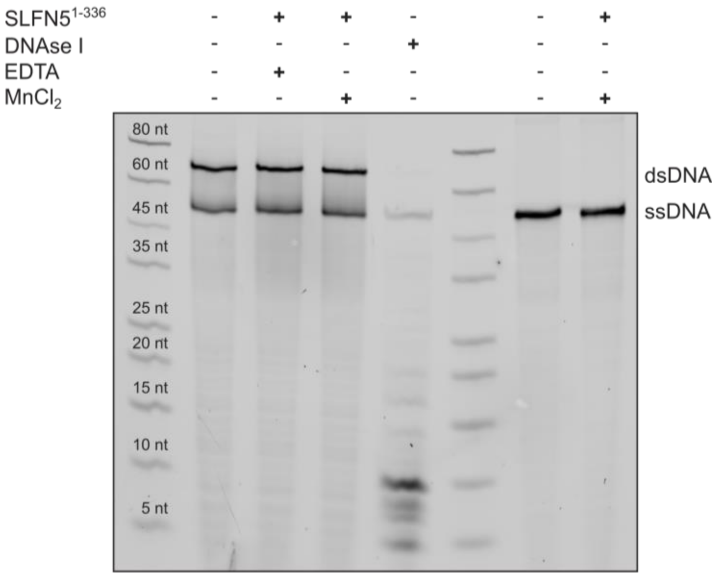

Supplementary Figure S11. Analysis of nuclease activity of SLFN5$^{1-336}$ on DNA. SLFN5$^{1-336}$ shows no nuclease activity on 50 bp dsDNA (lanes 2,3) and 50 nt ssDNA (lane 6). DNase I was used as positive control (lane 4).

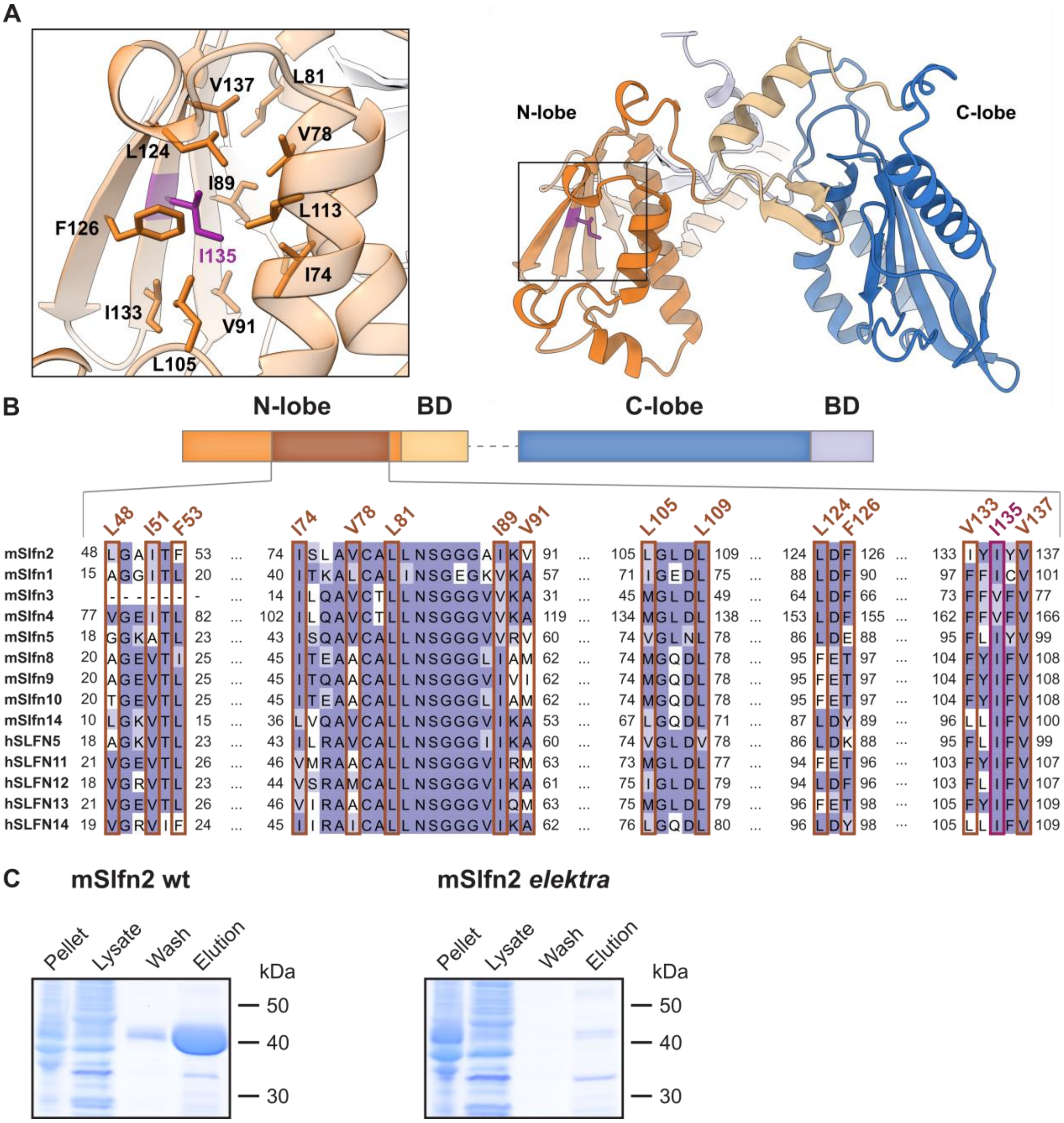

Supplementary Figure S12. Structural consequences of the *elektra* mutation (I135N) in mSlfn2. **(A)** AlphaFold model of full-length mSlfn2 (5) and close-up view of the hydrophobic patch region harboring the *elektra* mutation (I135, colored in purple). Amino acids are colored by heteroatom. **(B)** Schematic view of domain architecture of mSlfn2 and multiple sequence alignment of human and murine Schlafen family members. The conserved residues that form the hydrophobic core are highlighted in orange. **(C)** Ni-NTA purification of mSlfn2 wt and mSlfn2 *elektra* (I135N). Pellet, lysate, wash and elution fractions were subject to SDS-PAGE (stained with Coomassie blue). Similar volumes were loaded for both purifications. mSlfn2 has a molecular weight of approximately 42.5 kDa.

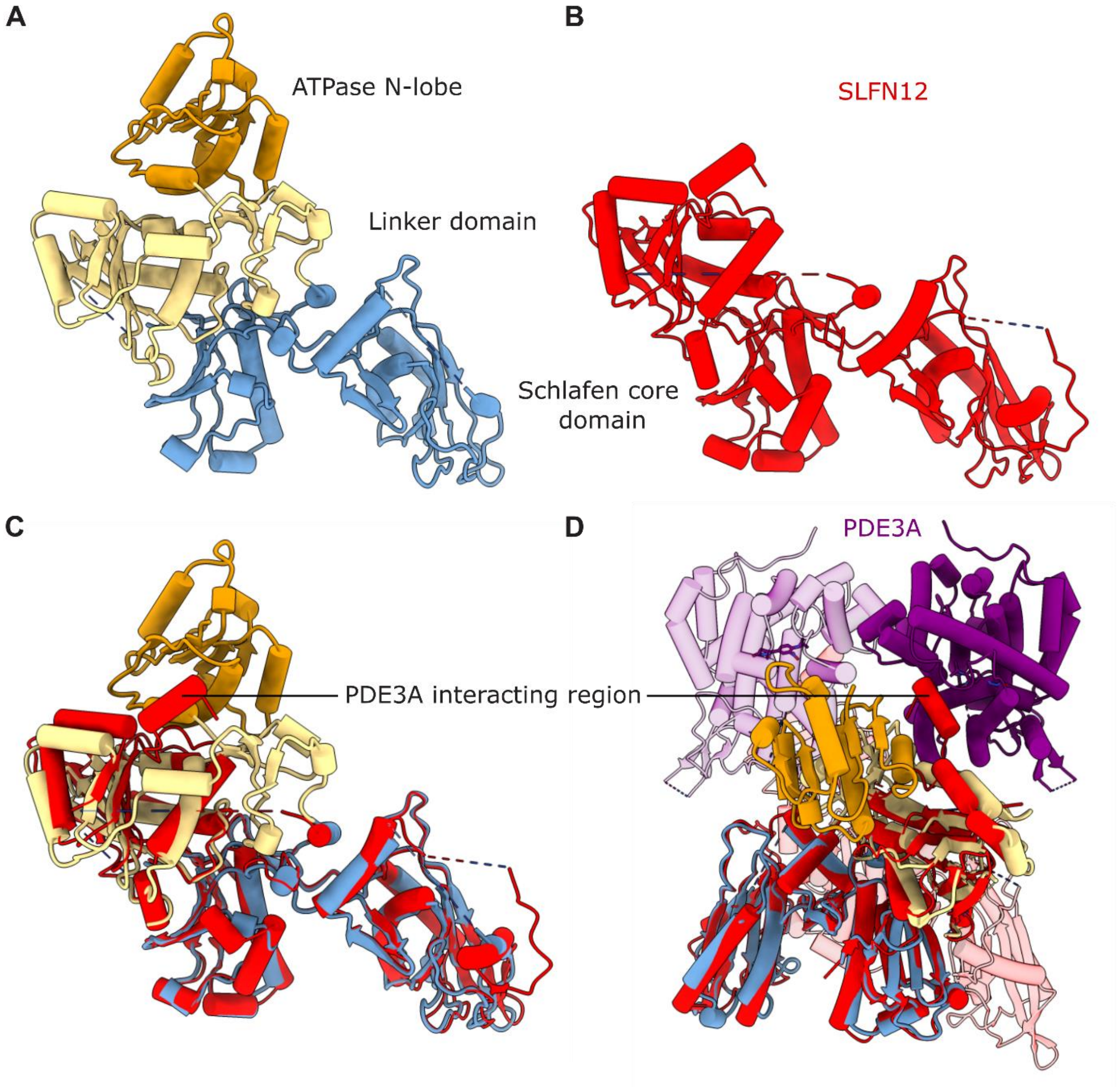

Supplementary Figure S13. Structural comparison of SLFN5 and SLFN12 (PDB: 7LRD). **(A)** Cartoon representation of SLFN5. The Schlafen core domain is depicted in blue, the linker domain in yellow and the helicase N-lobe in orange. **(B)** Cartoon representation of SLFN12 depicted in red (PDB: 7LRD). **(C)** Overlay of SLFN5 with SLFN12 (PDB: 7LRD). **(D)** Overlay of SLFN5 with the SLFN12-PDE3A complex (PDB: 7LRD). SLFN12 is depicted in red and PDE3A in purple.

Supplementary Table S1. Data collection, 3D reconstruction and refinement statistics

	H. sapiens SLFN5 dataset I	*H. sapiens* SLFN5 dataset II	*H. sapiens* SLFN5 dataset III
**Data collection and processing**			
**Magnification**	130 000	130 000	130 000
**Voltage (kV)**	300	300	300
**Electron exposure (e^-/Å^2)**	41.2	41.2	40.9
**Defocus range (µm)**	-1.1 to -2.9	-1.1 to -2.9	-1.1 to -2.9
**Pixel size (Å)**	1.046	1.046	1.046
**Tilt angle (°)**	0	25	25
**Symmetry imposed**	C1	C1	C1
**Initial particle images (no.)**	592 223	302 247	344 426
**Data processing (combined data)**			
**Final particle images (no.)**	140 715		
**Map resolution (Å)/ FSC threshold**	3.44/ 0.143		
**Refinement**			
**Initial model used (PDB code)**	6RI1		
**Model resolution (Å)**	3.7		
**FSC threshold**	0.5		
**Model resolution range (Å)**	3.2 -3.7		
**Map-sharpening *B* factor (Å^2)**	175.9		
**Model composition**			
**Nonhydrogen**	1 Zn		
**Protein residues**	643		
**R.m.s. deviations**			
**Bond lengths (Å)**	0.005		
**Bond angles (°)**	0.709		
**Validation**			
**MolProbity score**	2.19		
**Clashscore**	12.5		
**Poor rotamers (%)**	0.51		
**Ramachandran plot**			
**Favored (%)**	92.62		
**Allowed (%)**	7.38		
**Disallowed (%)**	0.0		
**PDB ID**	7PPJ		

Supplementary Table S2. Data collection and refinement statistics

**Data collection and processing**	**SLFN5$^{1\text{-}336}$**	**SLFN5$^{1\text{-}336}$**
**Wavelength [Å]**	1.0	1.28
**Space group**	P 1 2_1 1	P 3_2 2 1
**Unit cell dimensions**		
**a, b, c [Å]**	49.88, 64.04, 104.58	101.1, 101.1, 114.5
**α, β, γ [°]**	90, 93, 90	90, 90, 120
**Resolution range [Å]**	45.9 – 1.85 (1.92 – 1.85)	19.8 – 3.4
**R_{meas}**	9.0 (130.6)	15.21 (125.1)
**I/σI**	12.86 (1.43)	17.1 (2.2)
**Completeness [%]**	98.8 (97.6)	97.5 (83.7)
**Redundancy**	6.6 (6.3)	18.2 (14.9)
**Solvent content [%]**	44	72
**Matthews coefficient [A^3/Da]**	2.2	4.44
**Refinement**		
**Number reflections**	55695 (5454)	9144 (779)
**R_{work}/R_{free}**	0.18/0.21	0.21/0.26
**Number of atoms**		
**Protein**	4992	2483
**Ligands**	10	32
**Water**	289	
**Average B-factor [Å]**	42.361	64.22
**RMSD**		
**Bond lengths [Å]**	0.007	0.013
**Bond angles [°]**	0.85	1.41
**Ramachandran plot**		
**Favored [%]**	97.66	84.04
**Allowed [%]**	2.34	13.36
**Outliers [%]**	0.0	2.61
**PDB ID**	7Q3Z	6RR9

Supplementary Table S3. Oligonucleotides (5' to 3')

Purpose	Name	Sequence	Modification
Cloning	SLFN5_R271E_fwd	CATCATTTCTGCACACAGGAGCCTGAGATAAAATATGTC	
	SLFN5_R271E_rev	GACATATTTTATCTCAGGCTCCTGTGTGCAGAAATGATG	
	SLFN5_R326E_fwd	GACAATTGCCCACAGAAGAATGGACTGCTTG	
	SLFN5_R326E_rev	CAAGCAGTCCATTCTTCTGTGGGCAATTGTC	
	mSlfn2_I135N_fwd	GAGCGAAGGTTACATCTATAACTATGTTAAATCCTGGAGC	
	mSlfn2_I135N_rev	GCTCCAGGATTTAACATAGTTATAGATGTAACCTTCGCTC	
Biochemistry	5 dT	TTTTT	5'-FAM
	10 dT	TTTTTTTTTT	5'-FAM
	20 dT	TTTTTTTTTTTTTTTTTTTT	5'-FAM
	30 dT	TTTTTTTTTTTTTTTTTTTTTTTTTTTTTT	5'-FAM
	60 dT	TTTTTTTTTTTTTTTTTTTTTTTTTTTTTTTTTTTTTTTTTTTTTTTTTTTTTTTT TTTT	5'-FAM
	60bp_A/T_GC_fwd	TTTTTTTTTTTTTTTTTTTTTTTTTTTTTTTTTTTTTTTTTTTTTTTTTTGCTTTTT TTTT	5'-FAM
	60bp_A/T_GC_rev	AAAAAAAAAGCAAAAAAAAAAAAAAAAAAAAAAAAAAAAAAAAAAAAAAAAA AAAAAAAA	
	30 rU	UUUUUUUUUUUUUUUUUUUUUUUUUUUUUU	5'-FAM
	60 rU	UUUUUUUUUGCUUUUUUUUUUUUUUUUUUUUUUUUUUUUUUUUUUUUU UUUUUUUUUUUU	5'-FAM
	tRNA(Ser)	GUAGUCGUGGCCGAGUGGUUAAGGCGAUGGACUUGAAAUCCAUUG UGGUUUCCCCGCGCAGGUUCGAAUCCUGCCGACUACGCCA	5'-FAM
	41_nt_RNA_fwd	GACGGCC AUACCA CCC UGAACGGCCC GAUCUUGUCUGAUCC	5'-FAM
	41_nt_RNA_rev	GGAUCAGACAAGAUCGGGCCGUUCAGGGUGGUAUGGCCGUC	
	50_bp_DNA_fwd	AATTGGTCGTAGCAAGCTCTAGCACCGCTTAAACGCACGTACGCGCTGTC	5'-Cy5
	50_bp_DNA_rev	GACAGCGCGTACGTGCGTTTAAGCGGTGCTAGAGCTTGCTACGACCAATT	
	0N80 _fwd	CTGGAGAATCCCGGTGCCGAGG	5'-FAM
	0N80 _rev	TCGGTACCCGGGGATCCTCTAGAGTG	
	0N49 _fwd	CTGGAGAATCCCGGTGCCGAGG	
	0N49 _rev	TCGGAACACTATCCGACTGGCACC	5'-Cy5
	0N49/80 template	CTGGAGAATCCCGGTGCCGAGGCCGCTCAATTGGTCGTAGCAAGCTCTAG CACCGCTTAAACGCACGTACGCGCTGTCCCCCGCGTTTTAACCGCCAAGG GGATTACTCCCTAGTCTCCAGGCACGTGTCAGATATATACATCCTGTGCAT GTATTGAACAGCGACCTTGCCGGTGCCAGTCGGATAGTGTTCCGAGCTCC CACTCTAGAGGATCCCCGGGTACCGA	

REFERENCES

1. Punjani, A., Rubinstein, J.L., Fleet, D.J. and Brubaker, M.A. (2017) cryoSPARC: algorithms for rapid unsupervised cryo-EM structure determination. *Nat Methods*, **14**, 290-296.
2. Katoh, K., Misawa, K., Kuma, K. and Miyata, T. (2002) MAFFT: a novel method for rapid multiple sequence alignment based on fast Fourier transform. *Nucleic Acids Res*, **30**, 3059-3066.
3. Pettersen, E.F., Goddard, T.D., Huang, C.C., Meng, E.C., Couch, G.S., Croll, T.I., Morris, J.H. and Ferrin, T.E. (2021) UCSF ChimeraX: Structure visualization for researchers, educators, and developers. *Protein Sci*, **30**, 70-82.
4. Schneider, C.A., Rasband, W.S. and Eliceiri, K.W. (2012) NIH Image to ImageJ: 25 years of image analysis. *Nat Methods*, **9**, 671-675.
5. Jumper, J., Evans, R., Pritzel, A., Green, T., Figurnov, M., Ronneberger, O., Tunyasuvunakool, K., Bates, R., Žídek, A., Potapenko, A. *et al.* (2021) Highly accurate protein structure prediction with AlphaFold. *Nature*, **596**, 583-589.
