# Supplementary material for: Structural and biochemical characterization of human Schlafen 5
Source: Nucleic Acids Res. 2022 Jan 17;50(2):1147–61. doi: 10.1093/nar/gkab1278 (PMC8789055; doi:10.1093/nar/gkab1278)
Supplement: gkab1278_Supplemental_Files [file gkab1278_supplemental_files.zip › Supplementary Movie legends.docx]

**Supplementary Movie 1 legends**

Movie 1 illustrates the structural organization of human SLFN5, its proposed active- and nucleic acid binding site.
